# Development and Validation of the MAST ISOPLEX^®^ *VTEC* Kit for Simultaneous Detection of Shiga Toxin/Verotoxin 1 and 2 (*stx1/vt1 and stx2/vt2*) with Inhibition Control (IC) in a Rapid Loop-Mediated Isothermal Amplification (LAMP) Multiplex Assay

**DOI:** 10.3390/ijms251810067

**Published:** 2024-09-19

**Authors:** Monika Iwona Suwara, Matthew Bennett, Ilaria Anna Pia Voto, Christopher Allan Brownlie, Elizabeth Ann Gillies

**Affiliations:** 1 Mast Group Ltd., Mast House, Derby Rd, Bootle L20 1EA, UK; mbennet5@ed.ac.uk (M.B.); ivoto@mastgrp.com (I.A.P.V.); cbrownlie@mastgrp.com (C.A.B.); lgillies@mastgrp.com (E.A.G.); 2 Centre for Cardiovascular Science, University of Edinburgh, Edinburgh EH16 4TJ, UK

**Keywords:** loop-mediated isothermal amplification, MAST ISOPLEX^®^ *VTEC*, DNA/LYO3, lyophilization, multiplex, POC

## Abstract

Loop-mediated isothermal amplification (LAMP) is a cost-effective, rapid, and highly specific method of replicating nucleic acids. Adding multiple targets into a single LAMP assay to create a multiplex format is highly desirable for clinical applications but has been challenging due to a need to develop specific detection techniques and strict primer design criteria. This study describes the evaluation of a rapid triplex LAMP assay, MAST ISOPLEX^®^ *VTEC*, for the simultaneous detection of Shiga toxin/verotoxin 1 and 2 (*stx1/vt1* and *stx2/vt2*) genes in verotoxigenic *Escherichia coli (E. coli)* (*VTEC*) isolates with inhibition control (IC) synthetic DNA using a single fluorophore–oligonucleotide probe, MAST ISOPLEX^®^ *Probes,* integrated into the primer set of each target. MAST ISOPLEX^®^ *Probes* used in the MAST ISOPLEX^®^ *VTEC* kit produce fluorescent signals as they integrate with reaction products specific to each target, allowing tracking of multiple amplifications in real time using a real-time analyzer. Initial validation on DNA extracts from fecal cultures and synthetic DNA sequences (gBlocks) showed that the MAST ISOPLEX^®^ *VTEC* kit provides a method for sensitive simultaneous triplex detection in a single assay with a limit of detection (LOD) of less than 100 target copies/assay and 96% and 100% sensitivity and specificity, respectively.

## 1. Introduction

Loop-mediated isothermal amplification (LAMP) has one of the fastest amplification rates of all nucleic acid amplification tests (NAATs) whilst requiring only a single enzyme, Bst polymerase, which is capable of strand displacement amplification [1]. In the LAMP reaction, a minimum of four primers (F3, B3, FIP, and BIP) are required to act on an initial template sequence, though some variations may use additional primers such as loop primers. Preferably, two loop primers should be included in the LAMP primer mix to achieve the optimal reaction speed; however, since it is not always possible to design two individual loop primers due to the nature of the target sequence, LAMP can also be performed in the presence of a single loop primer. LAMP primers create a dumbbell structure allowing auto-cycling DNA replication. The dumbbell structures produce single-stranded loops which allow for Bst-mediated extension to displace downstream strands producing a cascade of amplification, creating a variety of looped DNA products [1]. The complexity of LAMP mechanics is contrasted by the simplicity of the testing protocols, which, together with the potential for highly specific and sensitive results, have helped make it the most implemented isothermal NAATs [2] and the one most ideally suited for integration into Point of Care (POC) devices [3].

Difficulty in single-vessel multiplexing for LAMPs is due to a tendency for cross-priming to occur when multiple primer sets are present at a high concentration requiring extra considerations for the already strict primer design criteria. In addition, LAMP detection has typically relied upon non-specific detection methods (e.g., turbidity, double-stranded DNA (dsDNA) intercalation, and colorimetric indicators) that cannot distinguish between targets in a multiplex scenario. Some of the literature has even concluded that LAMP cannot be used in multiplex formats [4,5]; however, Iseki et al. showed differentiation between two *Babesia* parasites by utilizing target-specific restriction endonuclease sites located in between primer hybridization sites. Amplicons could be identified based on the resulting fragment sizes seen after restriction digestion [6]. Pyrosequencing is another multiplex detection system that decodes tags inserted into the initiating LAMP primers after reaction completion to differentiate up to four targets simultaneously [7]. However, these post-amplification processing steps are undesirable as this increases the time to result and adds expense compared to real-time monitoring of LAMP.

Since the invention of LAMP in 2000 [1], numerous probe technologies compatible with this relatively novel method of isothermal nucleic acid detection have been described [6,8,9,10].

Zerilli et al. present probes that use quenching properties of a single nucleobase, guanine, which can absorb the light emission from a fluorophore linked to a complementary cytosine in the opposite DNA strand when the probe hybridizes with the target sequence [11]. Another system coined “detection of amplification by release of quenching” (DARQ) utilizes oligonucleotide-quencher probes to quench signals from Watson–Crick-related primer–fluorophore probes at the start of the assay [8]. Displacement from *Bst* extension during LAMP releases the specific quenching from this partnership, resulting in fluorescent signals, four of which could be detected simultaneously [8]. Further enhancement by conjugating the signaling fluorophore to a non-primer oligonucleotide leads to a reduction in the potential for primer–dimer LAMP to cause false positive DARQ signals [9].

DARQ probes [8] and molecular beacons [12] represent the two most convenient real-time multiplex technologies compatible with LAMP developed so far, with each mechanism having distinct advantages over the other. Low initial fluorescence baselines can be designed into both of these probe types through the choice of fluorophore–quencher pairings to create signals upon dissociation. However, both molecular beacons and DARQ probe-based tests require an additional double-labeled oligonucleotide, which increases the overall cost and complexity of the assay.

Guanine-quenching appears to rely on the redox properties of the fluorophore, which limits the pool of usable fluorophores during assay design [10]. The trade-off is the cost-benefit from having no need for a second fluorophore–oligonucleotide conjugant; however, some commonly used fluorophores like fluorescein are sensitive to pH decreases [13] in the reaction buffer, which may occur during LAMP product formation [14]. These pH fluctuations may result in an undesirable bias in the interpretation of results of LAMP assays relying on fluorescence quenching methods. A fluorescence increase is less ambiguous and can also be generated by adopting specific criteria such as conjugation position and nucleotide presence or absence in the surrounding environment [15], as demonstrated in a multiplex polymerase chain reaction scenario [16]. Such characteristics could be used in a LAMP multiplex to keep the number of required probes to one per target whilst potentially improving signal strength, sensitivity, and capacity for further targets.

Here, we describe a multiplex LAMP assay, MAST ISOPLEX^®^ *VTEC*, for the simultaneous detection of Shiga toxin/verotoxin 1 and 2 (*stx1/vt1* and *stx2/vt2*) genes in verotoxigenic *Escherichia coli (E. coli)* (*VTEC*) isolates with an inhibition control (IC) synthetic DNA using a single fluorophore–oligonucleotide probe, MAST ISOPLEX^®^ *Probes.* The fluorescent signal is generated by a fluorophore linked to an internally positioned cytosine and is enhanced when the probe becomes elongated by a *Bst*polymerase and is incorporated into the LAMP amplicon. MAST ISOPLEX^®^ *Probes* can be designed on a sequence of an existing LAMP primer and do not require a quenching mechanism, which makes them an easy alternative to other existing probe technologies demanding complex design and assay optimization.

The MAST ISOPLEX^®^ *VTEC* kit was validated using DNA extracts from a range of *vt1* and/or *vt2* positive and negative bacterial strains against a reference PCR assay method demonstrating 96% sensitivity and 100% specificity. The kit is presented in lyophilized format, can be shipped at ambient temperature, and displays long-term stability (over 2 years) at 2 °C to 8 °C, which eliminates the limitations related to cold chain shipment and long-term storage at minus 20 °C required for liquid reagents. The kit contains freeze-dried LAMP reaction pellets that dissolve immediately upon the addition of a reconstitution buffer (RB) and are presented in a format compatible with POC lab-on-a-chip devices.

## 2. Results

### 2.1. Test of Spectral Overlap between MAST ISOPLEX^®^ Probes in the MAST ISOPLEX^®^ VTEC Kit on the ABI7500 Fast Real-Time PCR System (Applied Biosystems)

**Figure 1:**
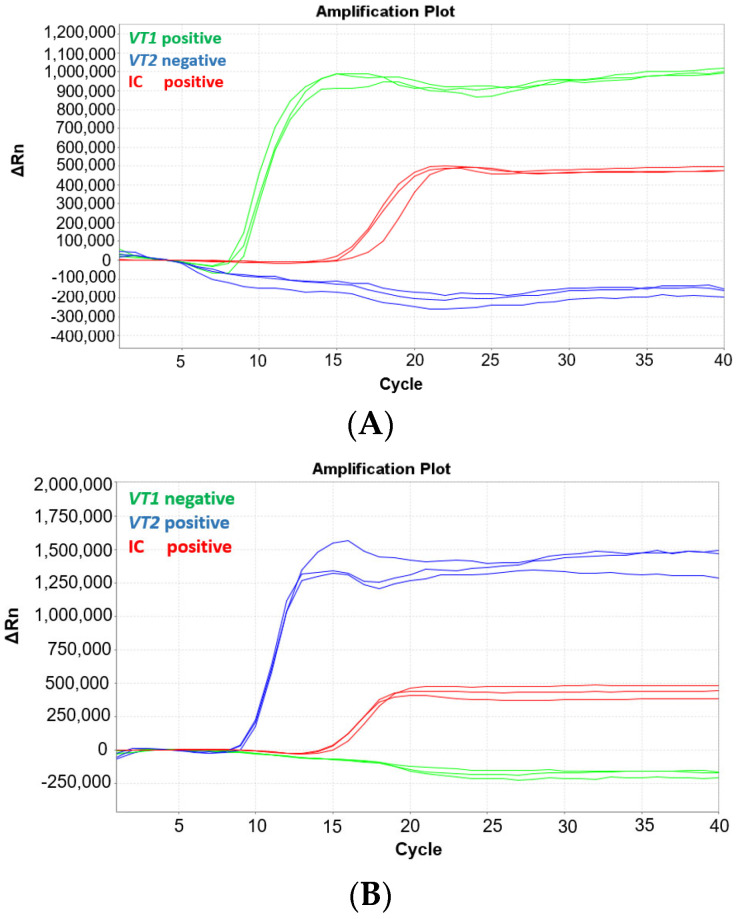

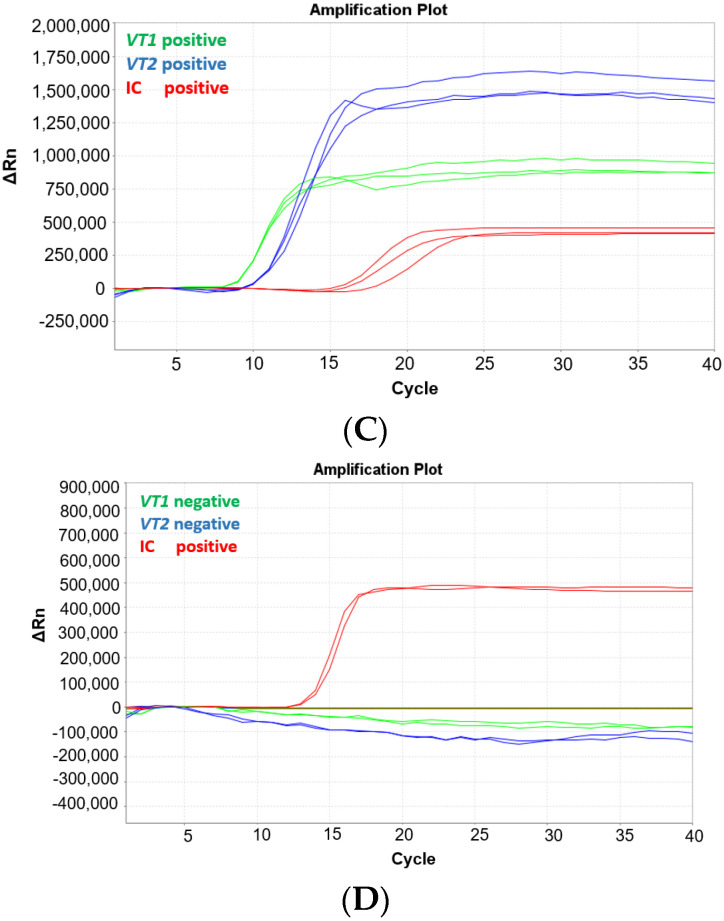
(**A**–**D**) Amplification plots generated on the ABI7500 Fast Real-Time PCR System with the MAST ISOPLEX^®^ *VTEC* kit using gBlocks containing *vt1* and *vt2* gene sequences. Amplification plots with gBlock sequences specific to (**A**) *vt1* (green line), (**B**) *vt2* (blue line), (**C**) *vt1* (green line) and *vt2* (blue line), and (**D**) IC only (red line). The IC control is indicated by a red line in Figure 1A–D. (positive—target DNA present; negative—target DNA absent). The *vt1* and *vt2* gBlocks were tested at 1 pg per reaction separately or in conjunction in the same reaction vessel. The tests were conducted in triplicate and internal control DNA (IC DNA) was included in each reaction.

The MAST ISOPLEX^®^ *VTEC* kit contains three fluorescent MAST ISOPLEX^®^ *Probes* conjugated with different fluorescent dyes: *vt1*-CY5, *vt2*-FAM, and IC-TAMRA. To ensure there is no spectral overlap between the probes, an experiment was conducted whereby the MAST ISOPLEX^®^ *VTEC* assay was tested on the ABI7500 Fast Real-Time PCR System (Applied Biosystems, Waltham, MA, USA) with gBlock sequences specific to either *vt1* or *vt2* primers (refer to Table S1). The *vt1* and *vt2* gBlocks were tested at 1 pg per reaction separately or in conjunction in the same reaction vessel. The tests were conducted in triplicate and internal control DNA (IC DNA) was included in each reaction. The MAST ISOPLEX^®^ *Probes* produced distinct amplification curves for each target DNA and no spectral overlap was observed between any of the tested fluorophores (CY5, FAM, or TAMRA). The results of the experiment are presented in Figure 1.

### 2.2. Test of the MAST ISOPLEX^®^ VTEC Kit on the Portable Mic qPCR Cycler (Bio Molecular Systems)

To evaluate if the MAST ISOPLEX^®^ *VTEC* kit is compatible with a portable Mic qPCR Cycler (Bio Molecular Systems, Upper Coomera, Queensland, Australia), which can be easily transported into low resource settings, the assay was tested on the Mic instrument using the positive control plasmid DNA available in the kit.

**Figure 2:**
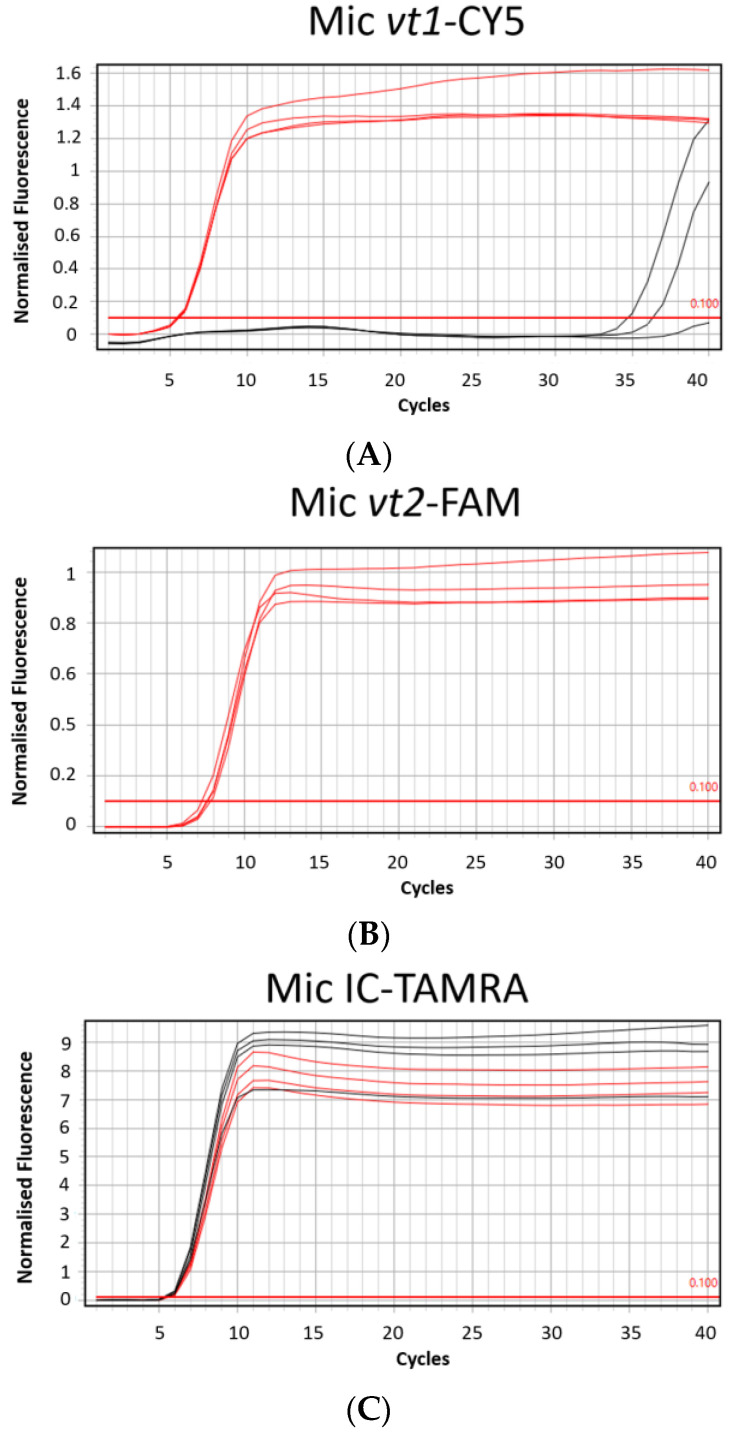
(**A**–**C**) Amplification plots generated on the Mic qPCR Cycler with positive control DNA plasmids from the MAST ISOPLEX^®^ *VTEC* kit. (**A**) CY5 channel (*vt1* target gene), (**B**) FAM channel (*vt2* target gene), (**C**) TAMRA (IC—inhibition control target sequence); Red lines—positive control DNA plasmids (PC); black lines—no template controls (NTCs). PC and NTCs were tested in 4 replicates.

The MAST ISOPLEX^®^ *Probes* present in the kit generated distinct amplification curves in each tested channel (FAM (green channel), TAMRA (orange channel), and CY5 (red channel)) and no spectral overlap was observed between any of the tested fluorophores (Figure 2A–C).

**Figure 3:**
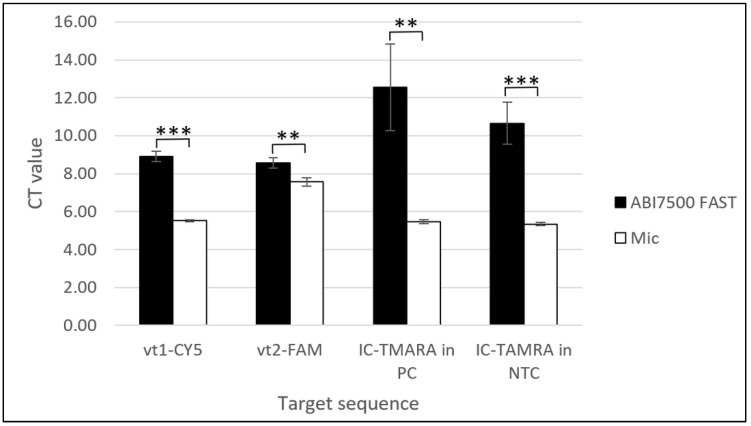
Comparison of mean Ct values obtained with positive control DNA plasmids (PC) from the MAST ISOPLEX^®^ *VTEC* kit using the ABI7500 Fast Real-Time PCR System and Mic qPCR Cycler. Black bars represent mean Ct values generated with the ABI7500 Fast Real-Time PCR System and white bars represent mean Ct values obtained with the Mic qPCR Cycler. Tests on the Mic qPCR Cycler were conducted in four replicates on the same batch of product and for the ABI7500 Fast Real-Time PCR System, data were collected from 3 separate experiments conducted on 3 evaluation batches of the MAST ISOPLEX^®^ *VTEC* kit. From each batch of the product, 3 individual LAMP reaction pellets were tested with positive control DNA plasmids (PC) or molecular grade water (NTC). Mean Ct values obtained with the ABI7500 Fast Real-Time PCR System and Mic qPCR Cycler were calculated for each target sequence (*vt1*-CY5, *vt2*-FAM, IC-TAMRA in PC (inhibition control in Positive Control samples) and IC-TAMRA in NTC (inhibition control in Positive control samples in No Template Control samples)). Statistical analysis was conducted using a Two-Sample t-Test with Excel software (Microsoft^®^, Redmond, WA, USA). The level of statistical significance is represented by the number of stars above the graphs (** *p* < 0.01, *** *p* < 0.001).

The amplification times of all the target genes, *vt1, vt2,* and IC, on the Mic qPCR Cycler (Bio Molecular Systems, Upper Coomera, Queensland, Australia) occurred before 10 min and were faster compared to Ct values obtained from the ABI7500 Fast Real-Time PCR System (Applied Biosystems, Waltham, MA, USA) by 1 (*vt2*-FAM) to 7 (IC-TAMRA in positive control (PC)) minutes depending on the target gene. Statistical analysis using a Two-Sample t-Test with Excel software (Microsoft^®^, Redmond, WA, USA) revealed that these differences were statistically significant for all target genes (*p* < 0.01 for *vt1*-CY5 and IC-TAMRA in PC and *p* < 0.001 for *vt2*-FAM and IC-TAMRA in no template controls (NTC)) (Figure 3).

No false amplification was detected on the ABI7500 Fast Real-Time PCR System (Applied Biosystems, Waltham, MA, USA) in NTCs before 50 min for any of the target genes. On the Mic qPCR Cycler (Bio Molecular Systems, Upper Coomera, Queensland, Australia), two false amplification results were noted in the CY5 channel at 34 and 36 min. 

The amplification plots obtained using positive control plasmids from the MAST ISOPLEX^®^ *VTEC* kit with the Mic qPCR Cycler (Bio Molecular Systems, Upper Coomera, Queensland, Australia) are demonstrated in Figure 2A–C. The comparison of Ct values obtained with the ABI7500 Fast Real-Time PCR System (Applied Biosystems, Waltham, MA, USA) and Mic qPCR Cycler (Bio Molecular Systems, Upper Coomera, Queensland, Australia) using positive control plasmids from the MAST ISOPLEX^®^ *VTEC* kit is shown in Figure 3.

### 2.3. Validation of the MAST ISOPLEX^®^ VTEC Kit on DNA Extracts from Clinical Specimens

The MAST ISOPLEX^®^ *VTEC* kit was evaluated using DNA extracts from 26 fecal samples grown on overnight cultures, which were kindly provided by Dr Claire Jenkins, Gastrointestinal Bacterial Reference Unit, Public Health England, London. The clinical samples were collected from patients who displayed symptoms of gastroenteritis and tested positive via qPCR for pathogenic *E. coli* expressing at least one of the following virulence factors: *stx1/vt1, stx2/vt2, haemolysin (hly), intimin (eae),* or an *E. coli O157* specific gene (for example *rfbE*). The results were compared against a reference method: BactoReal^®^ *E. coli* Typing *stx1* and the *stx2* (*STEC*) PCR multiplex kit from Ingenetix (Austria) to demonstrate that the performance is acceptable.

Of the 26 extracts tested by the reference method, 15 tested positive for *vt1*, 19 tested positive for *vt2* using multiplex PCR, 11 of these being co-infections, and 3 tested negative. The MAST ISOPLEX^®^ *VTEC* kit detected 13 *vt1* positive samples, 17 *vt2* positive samples, 9 co-infections, and 4 negative samples. Overall, 23 samples were positive for either the *vt1* or *vt2* gene when tested with the Ingenetix PCR kit and 22 samples were positive for at least one of the *vt* genes with the MAST ISOPLEX^®^ *VTEC* kit. The internal control (IC) DNA was successfully amplified in all samples. The results of the PCR and LAMP assays are summarized in Table 1(A,B). Clinical performance characteristics of the kit, i.e., sensitivity, specificity, Predictive Positive Value (PPV), Negative Predictive Value (NPV), and accuracy are shown in Table 2. The results demonstrated that the clinical sensitivity of the MAST ISOPLEX^®^ *VTEC* kit (combined data for *stx1* and *stx2* genes) is 96% compared to the PCR test. The specificity, PPV, NPV, and accuracy of the MAST ISOPLEX^®^ *VTEC* kit were found to be 100%, 100%, 75%, and 96%, respectively.

### 2.4. Analytical Specificity of the MAST ISOPLEX^®^ VTEC Kit

Assessment of the MAST ISOPLEX^®^ *VTEC* kit specificity was performed on DNA extracts from overnight cultures of 13 non-*vt1*/*vt2* expressing bacterial strains that could be found in fecal samples [17,18,19,20,21,22,23,24,25,26,27] and were available in-house at Mast Group Ltd. (refer to Table 3). Verotoxigenic *E. coli O157* was successfully amplified by the MAST ISOPLEX^®^*VTEC* kit and no cross-reactivity was observed with DNA extracts from any of the tested non-*vt1*/*vt2* expressing bacterial strains (Table 3). Data showed that the analytical specificity of MAST ISOPLEX^®^ *VTEC* is 100%. 

### 2.5. Validation of the MAST ISOPLEX^®^ VTEC Kit Using INSTAND e.V. Proficiency Samples

To assess the accuracy of the MAST ISOPLEX^®^ *VTEC* assay, the kit was tested with four proficiency samples obtained from INSTAND e.V. company (sample ID numbers: 1915341, 1915342, 1915343, and 1915344).

Proficiency testing of the MAST ISOPLEX^®^ *VTEC* kit showed that all results were valid and 2 out of 4 samples were positive for *VTEC* when tested with MAST ISOPLEX^®^ *VTEC* (Table 4A,B). The results were confirmed by the INSTAND e.V.’s certificate.

### 2.6. Analytical Sensitivity of the MAST ISOPLEX^®^ VTEC Kit

The analytical sensitivity test of the MAST ISOPLEX^®^ *VTEC* kit was performed with pEX-A128 plasmids with either *vt1* or *vt2* gene sequence inserts containing binding sites for *vt1* and *vt2* primers and probes used in the MAST ISOPLEX^®^ *VTEC* kit (Table S1). Probit analysis revealed that using MAST ISOPLEX^®^ *VTEC* kit components, the lower limit of detection at 95% Confidence Interval (CI) is 72.88 ± 2.6 (95% CI 18.46 to 1987.70) DNA copies/reaction and 19.07 ± 2.48 (95% CI 5.71 to 1081.16) DNA copies/reaction for *vt1* and *vt2* target sequences, respectively (Table 5A–D).

### 2.7. Stability of MAST ISOPLEX^®^ VTEC Reagents

To determine the shelf life of the MAST ISOPLEX^®^ *VTEC* kit, accelerated and initial real-time stability studies were conducted in accordance with the BS EN ISO 23640 standard. The raw data, result summary, acceptance criteria, storage conditions, and methodology used to conduct the simulated shipment study, accelerated, and real-time and in-use stability studies are included in the supporting information of Supplementary materials (refer to Figures S1–S8 and Tables S2–S14 and S20–S32).

Specification of acceptance criteria for the MAST ISOPLEX^®^ *VTEC* kit applied in the accelerated stability study is presented in Table S2. Temperature plots collected by data loggers recorded throughout the duration of the MAST ISOPLEX^®^ *VTEC* kit-accelerated stability study are shown in Figure S1.

The analysis of results from the accelerated stability study suggests that the active ingredients present in the MAST ISOPLEX^®^ *VTEC* kit maintain their activity for at least 13 weeks at 4 °C, 20 °C, 37 °C, and 50 °C (Figures S2–S5). According to the accelerated aging protocol formula, 13 weeks at 50 °C equates to a shelf life of 6.06 years at 4 °C or 13 months at 30 °C. However, the analysis of the influence of the kit storage temperature on the assay performance suggests that long-term storage at temperatures above 20 °C may have a negative impact on the kit specificity.

The in-depth analysis of raw data obtained during the accelerated stability study revealed that false amplification results before the assay cut-off point (40 min) may occur sporadically in random replicates and can be observed at any of the tested temperatures. 

Interestingly, the highest prevalence of false positive records before the assay cut-off point was detected with reagents exposed to 50 °C (7 out of 60 replicates, accounting for 11.6%) (Figure S6). 

Further data analysis revealed that there may be a temperature-dependent trend between the number of false positive results and the temperature storage of the kit. The kits exposed to 37 °C generated 6.66% false positive records (4 out of 60 replicates) and this percentage dropped to 5% (3 out of 60 replicates) when the reagents were stored at 20 °C. The lowest false positive results rate was observed with reagents stored at 4 °C, 3.33% (2 out of 60 replicates), translating into 96.67% assay specificity, which is within the expected range for the kit (>95% specificity) (Figure S7). Statistical analysis performed using the Pearson correlation test with Minitab^®^ Statistical Software v18 (Minitab^®^, LLC. Lock Haven, PA, USA) showed that the correlation coefficient, R, measuring a linear dependence between two storage temperatures and percentage of false positive results, was at 0.94, with *p* = 0.06.

A relationship between the MAST ISOPELX^®^ *VTEC* kit storage temperature and the number of false positive results in NTCs or the assay specificity is demonstrated in Figure S6 and Figure S7, respectively.

As the occurrence of false positive results seems to be random and not time-dependent and their prevalence exceeds the expected 5% mark only in kits stored at higher temperatures (37 °C and 50 °C), it has been decided to set the initial kit-claimed shelf life to 1 year at 2° to 8 °C. 

A real-time stability study showed that the product is stable for at least two years at the recommended 2 °C to 8 °C storage temperature (Table S8). Additionally, a simulated shipment study demonstrated that the kit can be shipped at ambient temperature without the loss of activity (Table S14). 

## 3. Discussion

This study describes the evaluation of the MAST ISOPLEX^®^ *VTEC* kit for simultaneous detection of Shiga toxin/verotoxin 1 and 2 (*stx1/vt1* and *stx2/vt2*) with an inhibition control (IC). 

MAST ISOPLEX^®^ *VTEC* is a rapid loop-mediated isothermal amplification (LAMP) multiplex assay using MAST ISOPLEX^®^ *Probes* technology allowing for the detection of multiple target sequences in a single reaction vessel. Although probe technologies for LAMP assays have been described before [6,8,9,10], MAST ISOPLEX^®^ *VTEC* is one of only a few multiplex probe LAMP assays currently available on the market.

In the presented study, the MAST ISOPLEX^®^ *VTEC* kit was challenged with DNA plasmids and gBlocks containing target sequences for *v1* and *vt2* genes, certified proficiency samples from INSTAND e.V. ring testing scheme, 13 DNA extracts from non-*v1* and *vt2* expressing bacterial strains, and 26 DNA extracts from fecal cultures obtained from patients displaying symptoms of acute gastroenteritis, which produced a range of Ct values (from 16.94 to 42.23) with a real-time PCR reference method.

The MAST ISOPLEX^®^ *VTEC* kit produced positive signals within 5–49.9 min with an analytical sensitivity of at least 100 DNA copies for both *vt1* and *vt2* plasmids alongside a simultaneous signal for IC at 1 pg/reaction. 

The results obtained with MAST ISOPLEX^®^ *VTEC* on all tested proficiency samples were confirmed by an INSTAND e.V. certificate demonstrating the assay accuracy.

The extensive literature search suggests that the MAST ISOPLEX^®^ *VTEC* kit has comparable performance to other isothermal *VTEC*-specific assays, with a sensitivity and specificity of 96% and 100%, respectively, indicating that LAMP-based MAST ISOPLEX^®^ *VTEC* product is a viable method for *VTEC* infection diagnosis [28,29,30,31,32].

The rapid and isothermal nature of LAMP along with the proprietary lyophilized format of the ‘pick and placeable’ multiplex LAMP pellets developed by Mast Group Limited makes these LAMP multiplex reagents compatible and easily transferable onto POC lab-on-a-chip diagnostic devices.

One of the desirable features of reagents developed for potential use in POC lab-on-a-chip diagnostic devices is long-term stability at ambient or refrigerator temperatures without a need for freezing. 

Data collected during real-time and shipping stability studies demonstrate that all reagents present in the MAST ISOPELX^®^ *VTEC* kit are stable for at least 2 years at 2 °C to 8 °C and that the kit can be shipped at ambient temperature without a loss of activity. The long-term storage conditions (2 °C to 8 °C) were chosen based on the analysis of the incidence of false positive results occurring before the assay cut-off point (40 min), which was lowest when the product was stored at 2 °C to 8 °C during the accelerated stability study (3.33% rate of false positive results in no template controls (NTCs) equating to 96.67% assay specificity). Tests on DNA extracts from clinical samples (overnight cultures) revealed that occasionally, positive samples may amplify between 40 and 50 min; therefore, it is recommended to confirm these late results with an alternative method such as PCR or traditional culture methods. 

MAST ISOPLEX^®^ *Probes* technology implemented in the MAST ISOPLEX^®^ *VTEC* kit allows for real-time detection of multiple targets in a single reaction without any post-amplification steps, providing a very simple and user-friendly solution to the existing probe technologies. 

MAST ISOPLEX^®^ *Probes* comprise a single oligonucleotide containing an internal cytosine labeled with a fluorophore of choice. The probes do not require any quenching mechanism and generate an increase in fluorescent signal when they hybridize to the target sequence and become elongated by *Bst* polymerase present in the LAMP reaction mix. Fluorescent outputs obtained with the MAST ISOPLEX^®^ *VTEC* kit on two separate real-time analyzers (ABI7500 Fast Real-Time PCR System (Applied Biosystems, Waltham, MA, USA) and Mic qPCR Cycler (Bio Molecular Systems, Upper Coomera, Queensland, Australia)) demonstrate that, despite a lack of a quenching mechanism, amplification curves generated with MAST ISOPLEX^®^ *Probes* are easily distinguishable and no spectral overlap is observed between any of the used fluorophores (FAM, TAMRA, and CY5). 

Multiplex LAMP assay methods without extra processing steps have been reported before [8,12]. In comparison to other single-fluorophore per target methods [11,16], the design of MAST ISOPLEX^®^ *Probes* is simplified by allowing dye conjugation to occur at an internal cytosine rather than requiring either a 5′ proximity to guanine [11] or the presence of a 3′ C or G, conjugation to a T within three bases of the 3′ end of the probe sequence, and the presence of a G in the surrounding three nucleotides [16]. LAMP primer design is complex and requires accounting for multiple criteria in length, positioning, 3′ free energy, and primer hybridization. This complexity escalates when using multiple primer sets simultaneously. MAST ISOPLEX^®^ *Probes* provide greater freedom at the probe design stage. In contrast to DARQ probes, which require an introduction to the LAMP reaction mix, additional oligonucleotides labeled with a dark quencher or a fluorophore are activated upon quencher/fluorophore dissociation. MAST ISOPLEX^®^ *Probes* function as additional primers that are activated upon elongation, making the assay design easier and potentially less prone to artifacts that may be caused by unspecific interactions between multiple oligonucleotides in a single reaction vessel. 

The reduced time to result and sensitivity and specificity of the *vt1*/*vt2* LAMP multiplex assay comparable to a standard PCR assay show that the MAST ISOPLEX^®^ *VTEC* kit using MAST ISOPLEX^®^ *Probes* technology may be suitable for clinical diagnostics. Due to its high specificity, the MAST ISOPLEX^®^ *VTEC* kit could also find application as a confirmatory tool to verify discrepant results obtained by other NAAT methods, which do not always provide 100% specificity when compared to culture methods or sequencing [33]. Due to high fluorescent signal intensities generated by MAST ISOPLEX^®^ *Probes* during DNA amplification, this technology could also be applied in digital LAMP [34], which is a semi-quantitative method using the compartmentalization of nucleic acids and LAMP reagents into individual droplets and, therefore, allowing for the quantification of an absolute number of the target nucleic acids in the sample.

Our study demonstrated that the MAST ISOPLEX^®^ *VTEC* kit with MAST ISOPLEX^®^ *Probes* is compatible with stationary real-time analyzers such as the ABI7500 Fast Real-Time PCR System (Applied Biosystems, Waltham, MA, USA) as well as the portable Mic qPCR Cycler (Bio Molecular Systems, Upper Coomera, Queensland, Australia), which can be easily transported into laboratories where sophisticated stationary PCR equipment is not available. Interestingly, the results obtained with the positive control plasmids with the Mic qPCR Cycler (Bio Molecular Systems, Upper Coomera, Queensland, Australia) were slightly (1 to 7 min) faster compared to the ABI7500 Fast Real-Time PCR System (Applied Biosystems, Waltham, MA, USA), offering a potential to reduce the assay cut-off point even further. The analysis of results obtained with NTCs revealed that on the Mic qPCR Cycler (Bio Molecular Systems, Upper Coomera, Queensland, Australia), nonspecific amplification with the MAST ISOPLEX^®^ *VTEC* kit is more likely to occur before 40 min in comparison to the ABI7500 Fast Real-Time PCR System (Applied Biosystems, Waltham, MA, USA), which produced specific results for up to 50 min during the quality control tests of three product evaluation batches. These results suggest that the fluorescent signal generated with MAST ISOPLEX^®^ *Probes* used in the MAST ISOPLEX^®^ *VTEC* kit can be successfully recognized with the portable Mic qPCR Cycler (Bio Molecular Systems, Upper Coomera, Queensland, Australia), but also, the assay cut-off point may need to be adjusted accordingly if the kit is to be used with this instrument.

The concentrations of MAST ISOPLEX^®^ *Probes* in the MAST ISOPLEX^®^ *VTEC* kit have been optimized to produce optimal fluorescent outputs with the ABI7500 Fast Real-Time PCR System (Applied Biosystems, Waltham, MA, USA) instrument featuring a complex optical system capable of detecting a broad spectrum of fluorophores. Other devices designed for low-cost isothermal amplification may be limited in the number and/or in the sensitivity of optical channels, and therefore, it is recommended to assess the compatibility of the MAST ISOPLEX^®^ *VTEC* kit with each real-time analyzer individually.

The MAST ISOPLEX^®^ *Probes*-based multiplex detection system applied in the MAST ISOPLEX^®^ *VTEC* kit is one of only a few existing methods allowing for simultaneous real-time detection of three target genes in LAMP without any post-reaction processing (e.g., gel electrophoresis, digestion with restriction enzymes, or pyrosequencing). Pyrosequencing is a different multiplex detection system that decodes tags but is not commonly used with LAMP; instead, it is more frequently applied in sequencing technologies. Nonetheless, a study by Liang et al. demonstrated how pyrosequencing can be utilized to distinguish between multiple targets in a multiplex LAMP reaction [7]. The advantage of MAST ISOPLEX^®^ *Probes* over other similar methods (e.g., DARQ probes or molecular beacons) is its cost-effectiveness and simplicity of design.

Results obtained with MAST ISOPLEX^®^ *Probes* implemented in the MAST ISOPLEX^®^ *VTEC* kit are recorded and analyzed in real time. The standard real-time LAMP assay duration is around 60 min; however, positive samples with high analyte concentrations can be detected within less than 10 min. This is in stark contrast to previously reported tests for Shiga toxin-producing Escherichia coli (STEC) detection that require post-amplification steps and combine PCR with either ELISA [35] or lateral flow assay [36], taking 6 and 2 h, respectively. 

Fluorescent probe-based multiplex LAMP assays are also much faster compared to traditional multiplex LAMP methods using digestion with restriction enzymes to distinguish between different LAMP products. These methods require around 15 min digestion steps followed by gel electrophoresis, which accounts for a further 40 to 90 min [37].

Despite significant advancements in the development of isothermal detection methods, one of the disadvantages of LAMP over traditional PCR methods remains limited multiplex capability. MAST ISOPLEX^®^ *VTEC* kit allows for the simultaneous detection of two target genes and internal control in a single reaction vessel. Further studies are required to investigate if the number of target sequences detected in a single LAMP reaction can be expanded without compromising the assay sensitivity. 

The cost per test with the MAST ISOPLEX^®^ *VTEC* kit is comparable with traditional multiplex PCR kits; however, its lyophilized format allows us to markedly reduce the cost of shipment. 

The assay reagents are presented in a lyophilized format compatible with point-of-care (POC) devices and can be transported without the need for a cold chain. The simplicity of the MAST ISOPLEX^®^ *Probes* design in combination with the outstanding features of LAMP, such as no requirement for thermal cycling, speed, and good specificity, and sensitivity makes the format of the MAST ISOPLEX^®^ *VTEC* kit easily transferable to low resource settings with limited access to expertise and complex equipment. 

## 4. Materials and Methods

### 4.1. DNA Extraction from Bacterial Cultures

DNA extracts from 26 overnight fecal cultures were kindly provided by Dr Claire Jenkins, Gastrointestinal Bacterial Reference Unit, Public Health England, London. The extracts were purified with the MagnaNA Pure extraction system (Roche, Basel, Switzerland) according to the manufacturer’s instructions. 

DNA from the non-*vt* expressing bacterial strains (obtained from MAST Group Ltd. cryobank) was extracted from cultures grown overnight on Columbia agar plates (PP0090, E&O Laboratories Limited, Burnhouse, Scotland) in a 35 °C incubator. Random colonies (5 to 10) from each plate were collected and resuspended in 1 mL of molecular-grade water (Sigma-Aldrich/Mreck Group St. Louis, MO, USA). The optical density of each suspension was adjusted to correspond to the 4 McFarland standard (equivalent to 12 × 10^8^ cfu/mL). The optical density of the bacterial suspensions was measured using a Densimat densitometer (Biomérieux, Craponne, France). DNA extraction from the bacterial suspensions was performed with a Seeprep12™ Na Common Kit using the SeePrep12™ automated magnetic bead nucleic acid extraction system (Seegene, Seoul, Republic of Korea) according to the manufacturer’s instructions. The yield of the extracted DNA was verified by the QuantiFluor^®^ dsDNA Kit (Promega, Madison, WI, USA) using a Quantus Fluorometer (Promega, Madison, WI, USA). 

### 4.2. DNA Synthetic Controls

*vt1* and *vt2* synthetic DNA controls were obtained in the form of gBlocks (Integrated DNA Technologies (IDT), Leuven, Belgium) or pEX-A128 plasmid vectors with inserted DNA sequences corresponding to the vt1 and vt2 gBlock sequences (Eurofins Genomics, Wolverhampton, UK). The vt1 and vt2 gBlocks were designed to contain primer-binding regions for the corresponding LAMP primers according to the following sequences available in GenBank: Escherichia *coli* O157:H7 *stx1* genes for Shiga toxin 1 variant A subunit, Shiga toxin 1 variant B subunit, complete cds, strain:AI2001/52 (Sequence ID: AB083044.1), and *Escherichia coli stx2* genes for Shiga-like toxin 2 A-subunit, Shiga-like toxin 2 B-subunit, complete cds (Sequence ID: AB030484.1), respectively. 

Inhibition control DNA (IC) is based on a random sequence generated with a Random DNA Sequence Generator (http://www.faculty.ucr.edu/~mmaduro/random.htm, accessed on 16 May 2014). The IC DNA control was constructed by Eurofins (UK) by inserting a 200-nucleotide long random sequence into a pEX-A128 expression plasmid. The sequences of gBlock controls used in the study are provided in Table S1.

### 4.3. Real-Time Polymerase Chain Reaction

A Real-Time Polymerase Chain Reaction on DNA extracts from clinical specimens was performed with the BactoReal^®^ *E. coli* Typing *stx1* and *stx2* (STEC) kit (Ref: DVEB04013) (Ingenetix, Wien, Austria). The BactoReal^®^ *E. coli* Typing kit is a multiplex PCR kit including primers and fluorescent probes specific to *stx1/vt1* (FAM channel) or *stx2/vt2* (VIC/HEX channel) and an internal control DNA (CY5 channel). The kit is compatible with real-time analyzers such as the ABI7500 Fast Real-Time PCR System (Applied Biosystems, Waltham, MA, USA), LightCycler^®^ 480 (Roche, Basel, Switzerland), and Mx300P^®^ (Agilent, Santa Clara, CA, USA) and can be used with DNA purified from fecal samples and bacterial cultures. The detection of *vt1* and *vt2* genes in DNA extracts from fecal clinical cultures obtained from PHE London was performed using the BactoReal^®^ *E. coli* Typing kit as per the manufacturer’s instruction for use (IFU). In total, 5 µL of purified DNA sample or molecular grade water for no template control (NTC) was added to 15 µL of reaction mix loaded into a 96-well plate and the assay was run on an ABI7500 Fast Real-Time PCR System (Applied Biosystems, Waltham, MA, USA). Samples that produced a positive signal in the FAM channel were considered positive for the *vt1* gene and samples that produced a positive signal in the VIC/HEX channel were considered positive for the *vt2* gene. 

### 4.4. Loop-Mediated Isothermal Amplification (LAMP) Using the MAST ISOPLEX^®^ VTEC Kit

The detection of *vt1* and *vt2* genes of verotoxigenic *Escherichia coli* was performed using the MAST ISOPLEX^®^ *VTEC* LAMP multiplex assay (DNA/LYO3) (Mast Group Ltd., Bootle, UK) as per the instruction for use (IFU). The kit contains lyophilized (freeze-dried) multiplex LAMP pellets (PEL1) and vacuum-dried primer–probe mixes (PP1). Prior to use, the LAMP pellet (PEL1) was reconstituted in 20 µL of reconstitution buffer (RB) and the primer–probe pellet (PP1) was resuspended in 10 µL of molecular grade water (WTR). Each LAMP reaction was set up using 2 µL of reconstituted LAMP master mix, 1 µL of reconstituted primer probe mix (PP1), 2 µL of molecular grade water (WTR), and 5 µL of DNA sample (DNA extract from overnight cultures, gBlocks, or DNA plasmids) or molecular grade water for no template controls (NTC). 

The LAMP assays were performed using either a stationary ABI7500 Fast Real-Time PCR System (Applied Biosystems, Waltham, MA, USA) or a portable Magnetic Induction Cycler (Mic) qPCR Cycler (Bio Molecular Systems, Upper Coomera, Queensland, Australia). 

The LOD studies, analytical specificity studies, clinical performance studies, proficiency testing, and stability studies were conducted on the ABI7500 Fast Real-Time PCR System (Applied Biosystems, Waltham, MA, USA).

All tests conducted with the ABI7500 Fast Real-Time PCR System (Applied Biosystems, Waltham, MA, USA) were set up in 96 well plates (Applied Biosystems, Waltham, MA, USA) and were run at a constant temperature of 63 °C for 60 min. 

The compatibility of the MAST ISOPLEX^®^*VTEC* kit with the portable Mic qPCR Cycler (Bio Molecular Systems, Upper Coomera, Queensland, Australia) was performed in 0.1 mL tube strips containing a layer of proprietary oil, preventing the samples from evaporation throughout the duration of the test. The test was performed as per the IFU using Positive Control DNA: PC (VT1VT2), containing a mix of plasmids with inserted sequences specific to *vt1* and *vt2* genes. The assay on the Mic qPCR Cycler (Bio Molecular Systems, Upper Coomera, Queensland, Australia) was run for 40 min at a constant temperature of 63 °C. 

The results on both instruments were visualized in TAMRA, FAM, and CY5 channels and the time to amplification was determined based on Ct values (threshold cycle).

### 4.5. Test of MAST ISOPLEX^®^ VTEC Kit on INSTAND e.V Proficiency Samples

Proficiency testing of MAST ISOPLEX^®^ *VTEC* was performed on four lyophilized samples provided by the INSTAND e.V. company (Düsseldorf, Germany), as part of a proficiency testing program. Sample pellets were dissolved in sterile molecular high-grade water (Sigma-Aldrich/Mreck Group St. Louis, MO, USA), as per the manufacturer’s instruction. When pellets are completely dissolved, the resulting suspension is considered a native clinical specimen according to the manufacturer, with the sample matrix containing proteins, bacteria, and human cells alongside the target organism. DNA was extracted with the Maga Zorb^®^ DNA Mini-Prep kit from (Promega, Madison, WI, USA) and tested with the MAST ISOPLEX^®^ *VTEC* kit. Each sample was tested in triplicate. The assay was set up as per IFU and was run for 60 min at 63 °C with FAM/TAMRA/Cy5 channels using the ABI7500 Fast Real-Time PCR System (Applied Biosystems, Waltham, MA, USA).

### 4.6. Determination of the Analytical Sensitivity of the MAST ISOPLEX^®^ VTEC Kit

The analytical sensitivity test of the MAST ISOPLEX^®^ *VTEC* kit was performed with pEX-A128 plasmids with either the *vt1* or *vt2* gene sequence insert containing binding sites for *vt1* and *vt2* primers and probes used in the MAST ISOPLEX^®^ *VTEC* kit (Table S1). The plasmids were obtained from Eurofins Genomics (Wolverhampton UK). The MAST ISOPLEX^®^ *VTEC* triplex assay was tested with serial dilutions of *vt1* and *vt2* control plasmids ranging from 0.5 fg to 0.00005 fg per reaction. Each plasmid concentration was tested in 9 replicates. The assays were set up as per the kit’s IFU. All tests were run on the ABI7500 Fast Real-Time PCR System (Applied Biosystems, Madison, WI, USA) for 60 min at a constant temperature of 63 °C. Samples amplifying below 40 min were considered positive.

Plasmid concentrations were converted into DNA copy numbers based on their size (3419 bp for *vt1* plasmid and 3439 bp for *vt2* plasmid) using an online ‘DNA Copy Number and Dilution Calculator’ (Thermo Fisher Scientific, Loughborough, UK):

DNA Copy Number Calculator | Thermo Fisher Scientific—UK.

The number of positive events per analyte’s concentration was used in Probit Analysis to determine the limit of detection of each target in a multiplex assay. Probit analysis was performed using Minitab^®^ Statistical Software v18 (Minitab^®^, LLC., Lock Haven, PA, USA). 

### 4.7. Acceptance/Rejection Criteria

Samples were considered positive if they generated a positive amplification curve (Ct value) in either CY5 (for *vt1* /*stx1* gene) or FAM (*vt2*/*stx2* gene) channels (single positives) or in both channels (double positives) before 40 min. Samples were considered negative if no amplification curve occurred in either the FAM or CY5 channel but a positive signal was generated by the internal control (IC) in the TAMRA channel. If no amplification of the internal control was detected in the TAMRA channel, the result was considered invalid. A positive control (*vt1*/*vt2* plasmid mix provided in the kit) and a negative control (molecular grade water) were included in each experimental setup. Results that amplified between 40 and 50 min in unknown samples were considered positive after confirmation with an alternative method such as real-time PCR. The result interpretation criteria are summarized in Table 6.

### 4.8. Statistical Analysis

The assay limit of detection was determined using Probit analysis with Minitab^®^ statistical software v18 (Minitab^®^, LLC., Lock Haven, PA, USA).

Correlation analysis was performed using the Pearson correlation test with Minitab^®^ Statistical Software v18 (Minitab^®^, LLC., Lock Haven, PA, USA).

A comparison of Ct values obtained with the MAST ISOPLEX^®^ *VTEC* kit using the ABI7500 Fast Real-Time PCR System (Applied Biosystems, Waltham, MA, USA) and Mic qPCR Cycler (Bio Molecular Systems, Upper Coomera, Queensland, Australia) was conducted using a Two-Sample t-Test with Excel software 2016 (16.0.5465.1000), (Microsoft^®^, Redmond, WA, USA).

Calculation of the clinical performance characteristics of the kit, i.e., sensitivity, specificity, Predictive Positive Value (PPV), Negative Predictive Value (NPV), and accuracy, was performed using the following formulas:

(1) $$Sensitivity=\frac{No.\;of\;true\;positives}{No.\;of\;true\;positives+No.\;of\;false\;negatives}$$

(2) $$Specificity=\frac{No.\;of\;true\;negatives}{No.\;of\;true\;negatives+No.\;of\;false\;positives}$$

(3) $$PPV=\frac{No.\;of\;true\;positives}{No.\;of\;true\;positives+No.\;of\;false\;positives}$$

(4) $$NPV=\frac{No.\;of\;true\;negatives}{No.\;of\;true\;negatives+No.\;of\;false\;negatives}$$

(5) $$Accuracy=\frac{\left(No.\;of\;true\;positives+No.\;of\;true\;negatives\right)}{\left(No.\;of\;true\;positives+No.\;of\;true\;negatives+No.\;of\;false\;positives+No.\;of\;false\;negatives\right)}$$

The shelf life based on data derived from accelerated testing conditions was determined using the following accelerated ageing protocol formula:

R = 2^A^X

where

R = real time equivalent

X = actual time under accelerated conditions 

A = Increase in temperature over normal storage temperature (°C)/10

## 5. Patents

A full specification of MAST ISOPLEX^®^ *Probes* is included in patent publication number WO/2015/063498. 

## Figures and Tables

**Table 1:** (**A**) Validation of the MAST ISOPLEX^®^ *VTEC* kit on DNA extracts from cultured clinical specimens. (**B**) Interpretation of the MAST ISOPLEX^®^ *VTEC* kit LAMP assay results on DNA extracts from cultured clinical specimens.

(A)						
Sample Number	*vt1* Multiplex PCR Result	*vt1* Multiplex LAMP Result	*vt2* Multiplex PCR Result	*vt2* Multiplex LAMP Result	*vt1* or *vt2* Positive (Combined PCR Results)	*vt1* or *vt2* Positive (Combined LAMP Results)
1	39.61	ND	42.23	49.9	+	+
2	29.59	30.57	30.07	48.44	+	+
3	ND	ND	24.91	26.71	+	+
4	30.69	31.51	30.63	27.31	+	+
5	ND	ND	36.53	44.2	+	+
6	29.59	30.53	30.28	46.88	+	+
7	ND	ND	16.94	10.12	+	+
8	27.13	30.64	27.49	36.65	+	+
9	34.97	38.91	ND	ND	+	+
10	ND	ND	ND	ND	−	−
11	ND	ND	37.73	ND	+	−
12	ND	ND	23.84	14.26	+	+
13	34.39	28.66	34.38	28.94	+	+
14	38.3	ND	40.22	28.73	+	+
15	36.12	39.8	ND	ND	+	+
16	ND	ND	ND	ND	−	−
17	31.74	28.1	ND	ND	+	+
18	28.28	30.43	24.89	16.92	+	+
19	23.18	24.16	23.68	33.21	+	+
20	36.31	37.62	33.78	ND	+	+
21	ND	ND	ND	ND	−	−
22	25.43	28.38	ND	ND	+	+
23	ND	ND	26.12	19.78	+	+
24	ND	ND	22.16	27.29	+	+
25	28.74	30.06	28.54	34.92	+	+
26	ND	N/A	29.2	18.78	+	+
**(B)**						
Number of true positive results				22		
Number of false positive results				0		
Number of true negative results				3		
Number of false negative results				1		

Validation of the MAST ISOPLEX^®^ *VTEC* kit using DNA extracts from 26 fecal samples grown on overnight cultures. Raw data from the multiplex PCR assay (BactoReal^®^ E. coli Typing *stx1* and *stx2* (STEC) PCR multiplex kit from Ingenetix) and MAST ISOPLEX^®^ *VTEC* kit LAMP assay are shown. ND, not detected; +, sample is positive for either or both vt1/vt2 targets; −, sample is negative for both vt1/vt2 targets.

**Table 2:** Clinical performance of the MAST ISOPLEX^®^ *VTEC* kit.

Clinical Performance Indicator	Calculation	Result	Result [%]
Sensitivity	22/(22 + 1)	0.96	96%
Specificity	3/(3 + 0)	1	100%
PPV	22/(22 + 0)	1	100%
NPV	3/(3 + 1)	0.75	75%
Accuracy	(22 + 3)/(22 + 3 + 1 + 0)	0.96	96%

PPV, Positive Predictive Value; NPV, Negative Predictive Value.

**Table 3:** Assessment of the MAST ISOPLEX^®^ *VTEC* Kit Specificity.

Bacteria Species	Origin	ID Number	MAST ISOPLEX^®^ *VTEC* LAMP Assay Results (Ct Values)			Results Interpretation
			*vt1*	*vt2*	IC DNA	
*Escherichia coli O157*	unknown	unknown	7.4	8.45	15.74	Valid. Positive for verotoxigenic *E. coli*
No template control	N/A	N/A	ND	ND	12.18	Valid. Negative for verotoxigenic *E. coli*
*Escherichia coli*	ATCC	11,303	ND	ND	12.26	Valid. Negative for verotoxigenic *E. coli*
*Edwardsiella tarda*	NCTC	11,934	ND	ND	12.07	Valid. Negative for verotoxigenic *E. coli*
*Clostridium sporogenes*	ATCC	19,404	ND	ND	12.4	Valid. Negative for verotoxigenic *E. coli*
*Proteus mirabilis*	ATCC	29,906	ND	ND	12.27	Valid. Negative for verotoxigenic *E. coli*
*Vibrio parahaemolyticus*	ATCC	17,802	ND	ND	12.25	Valid. Negative for verotoxigenic *E. coli*
*Peptostreptococcus anaerobius*	ATCC	27,337	ND	ND	12.39	Valid. Negative for verotoxigenic *E. coli*
*Bacteroides fragilis*	ATCC	25,285	ND	ND	12.24	Valid. Negative for verotoxigenic *E. coli*
*Citrobacter freundii*	ATCC	8090	ND	ND	15.59	Valid. Negative for verotoxigenic *E. coli*
*Proteus hauseri*	ATCC	13,315	ND	ND	12.41	Valid. Negative for verotoxigenic *E. coli*
*Pseudomonas aeruginosa*	ATCC	12,903	ND	ND	12.29	Valid. Negative for verotoxigenic *E. coli*
*Staphylococcus aureus*	ATCC	9144	ND	ND	11.64	Valid. Negative for verotoxigenic *E. coli*
*Enterobacter cloacae*	NCTC	11,936	ND	ND	12.22	Valid. Negative for verotoxigenic *E. coli*
*Yersinia enterocolitica*	ATCC	9610	ND	ND	12.15	Valid. Negative for verotoxigenic *E. coli*

MAST ISOPLEX^®^ *VTEC* kit LAMP assay raw data of each target for each sample are shown, alongside assay results interpretation. ND—not detected.

**Table 4:** (**A**) Validation of the MAST ISOPLEX^®^ *VTEC* kit on INSTAND e.V. proficiency samples. (**B**) Interpretation of the MAST ISOPLEX^®^ *VTEC* kit results on INSTAND e.V. proficiency samples.

(A)												
Sample No./Target	Sample 1D: 19153											
	41 n1	41 n2	41 n3	42 n1	42 n2	42 n3	43 n1	43 n2	43 n3	44 n1	44 n2	44 n3
*vt1*	13.28	12.46	11.97	ND	ND	ND	ND	ND	ND	ND	ND	ND
*vt2*	24.31	31.94	23.32	ND	ND	ND	16.62	18.04	17.45	ND	ND	ND
IC	11.67	11.80	12.01	11.84	11.56	11.73	11.81	11.68	11.69	11.57	11.76	11.88
**(B)**												
**SAMPLE ID**	***vt1* Target Gene**			***vt2* Target Gene**			**IC Target**			**Result**		
1915341	+ (3/3 replicates)			+ (3/3 replicates)			+ (3/3 replicates)			Valid. The sample is positive for *vt1 and vt2.*		
1915342	− (3/3 replicates)			− (3/3 replicates)			+ (3/3 replicates)			Valid. The sample is negative for *vt1 and vt2.*		
1915343	− (3/3 replicates)			+ (3/3 replicates)			+ (3/3 replicates)			Valid. The sample is positive for *vt2 only*.		
1915344	− (3/3 replicates)			− (3/3 replicates)			+ (3/3 replicates)			Valid. The sample is negative for *vt1 and vt2*.		

(**A**) Ct values obtained in a validation experiment of the MAST ISOPLEX^®^ *VTEC* kit on INSTAND e.V. proficiency samples. n—replicate number; ND, not detected; IC, inhibition control. (**B**) + the targets amplified; − the targets did not amplify.

**Table 5:** (**A**) Input data used for the calculation of the limit of detection (LOD) of the *vt1* assay of the MAST ISOPLEX^®^ *VTEC* kit with Probit analysis. (**B**) Input data used for the calculation of the limit of detection (LOD) of the *vt2* assay of the MAST ISOPLEX^®^ *VTEC* kit with Probit analysis. (**C**) Results of Probit analysis performed to determine the LOD of the *vt1* assay of the MAST ISOPLEX^®^ *VTEC* kit. (**D**) Results of Probit analysis performed to determine the LOD of the *vt2* assay of the MAST ISOPLEX^®^ *VTEC* kit.

(A)				
Concentration of *vt1* Plasmid Per Reaction	Number of Copies of *vt1* Plasmid Per Reaction	Total Number of Replicates	Number of Positive Replicates Obtained with *vt1* Plasmid	Number of Positive Replicates Obtained with IC (Internal Control Plasmid)
0.5 fg	135.45	9	9	9
0.1 fg	27.09	9	8	9
0.005 fg	1.3545	9	1	9
0.001 fg	0.2709	9	2	9
0.00005 fg	0.013545	9	0	9
**(B)**				
**Concentration of *vt2* Plasmid Per Reaction**	**Number of Copies of *vt2* Plasmid Per Reaction**	**Total Number of Replicates**	**Number of Positive Replicates Obtained With *Vt2* Plasmid**	**Number of Positive Replicates Obtained with IC (Internal Control Plasmid)**
0.5 fg	134.7	9	9	9
0.1 fg	26.94	9	9	9
0.005 fg	1.347	9	2	9
0.001 fg	0.2694	9	1	9
0.00005 fg	0.01347	9	0	9
**(C)**				
***vt1* Plasmid**	**Percentile**	**Error**	**Lower**	**Upper**
DNA copies per reaction	72.88	2.60	18.46	1987.70
**(D)**				
***vt2* Plasmid**	**Percentile**	**Error**	**Lower Limit**	**Upper Limit**
DNA copies per reaction	19.07	2.48	5.71	1081.16

**Table 6:** Interpretation of the results obtained with the MAST ISOPLEX^®^ *VTEC* kit.

Controls/Samples	Target Gene (*vt1-* CY5 or/ and *vt2-* FAM)	IC TAMRA	Interpretation
Negative control	−	+	Valid
Positive control	+	+	Valid
Sample	−	+	Valid; negative for the target gene
Sample	−	−	Invalid
Sample	+	+/− *	Valid; positive for the target gene

* Inhibition control (IC TAMRA) can occasionally fail to amplify if target DNA is present in extremely high quantities, out-competing IC TAMRA primers and probes for reagents (Ref. MAST ISOPLEX^®^ *VTEC* IFU).

## Data Availability

The original contributions presented in the study are included in the article/supplementary material; further inquiries can be directed to the corresponding author.

## Supplementary Materials

The following supporting information can be downloaded at: https://www.mdpi.com/article/10.3390/ijms251810067/s1.
