# Supplementary material for: Development and Validation of the MAST ISOPLEX® VTEC Kit for Simultaneous Detection of Shiga Toxin/Verotoxin 1 and 2 (stx1/vt1 and stx2/vt2) with Inhibition Control (IC) in a Rapid Loop-Mediated Isothermal Amplification (LAMP) Multiplex Assay
Source: Int J Mol Sci. 2024 Sep 19;25(18):10067. doi: 10.3390/ijms251810067 (PMC11432264; doi:10.3390/ijms251810067)
Supplement: Supplementary file 1 [file ijms-25-10067-s001.zip › ijms-3184491-supplementary.pdf]

International Journal of *Molecular Sciences*

MDPI

Article

Development and Validation of the MAST ISOPLEX® *VTEC* Kit for Simultaneous Detection of Shiga Toxin/Verotoxin 1 and 2 (*stx1*/*vt1 and stx2*/*vt2*) with Inhibition Control (IC) in a Rapid Loop-Mediated Isothermal Amplification (LAMP) Multiplex Assay

Monika Iwona Suwara [1,*], Matthew Bennett [1,2], Ilaria Anna Pia Voto [1], Christopher Allan Brownlie [1] and Elizabeth Ann Gillies [1]

[1] Mast Group Ltd., Mast House, Derby Rd, Bootle, Merseyside, L20 1EA, UK; msuwara@mastgrp.com(M.I.S.) ivoto@mastgrp.com(I.A.P.V.); cbrownlie@mastgrp.com(C.A.B); lgillies@mastgrp.com(E.A.G.)
[2] Centre for Cardiovascular Science, University of Edinburgh, Edinburgh EH16 4TJ, UK; mbennet5@ed.ac.uk(M.B.)
* Correspondence: msuwara@mastgrp.com

Appendix A

Supporting information: .

1. Abbreviations:

ΔRn	change in reporter fluorescence normalized to a baseline level of fluorescence
Bst	large fragment of the *Bacillus stearothermophilus* DNA polymerase
Ct	threshold cycle
DARQ	detection of amplification by release of quenching
dsDNA	double-stranded deoxyribonucleic acid
IC	inhibition control
IFU	instruction for use
LAMP	loop-mediated isothermal amplification
LOD	limit of detection
Mic	Magnetic induction cycler
NAATs	nucleic acid amplification tests
ND	not detected
NTC	no template control
PCR	polymerase chain reaction
PEL 1	multiplex LAMP pellet
PHE	Public Health England
POC	Point of Care
PP1	primer probe mix
RB	reconstitution buffer
rfbE	O-antigen synthesis gene
STEC	Shigatoxigenic *Escherichia coli*
stx1	Shiga-like toxin 1
stx2	Shiga-like toxin 2
vt1	verotoxin 1
vt2	verotoxin 2

VTEC	verotoxigenic *Escherichia coli*
SD	standard deviation
n	number of replicates
NTC	no template control
ND	not detected
Ct	threshold cycle

2. Gene sequences used in the study

Table S1. IC (inhibition control), *vt1* and *vt2* gene sequences inserted into the pEX-A128 plasmid vector used in the study.

Name of the DNA sequence	DNA Sequence
IC	AGCCCACGGAGACCACTGACCGATCTACCTGAACGGCGACCATCTGTGTGGTACTGGGGCG-<br>GAGAGATAACTACGGTGCCGCTTACAGCCCCTCTGTCGTCGCTGACGTCTGTAGTCTAGCCT<br>CATTATGATTGTACGCTATTCAGGGATTGACTGATACCGGAAGA-<br>CATCTCAAATGAAGTGGTCTATGCGACAGAGAC
VT1	ATGAAAATAATTATTTTTAGAGTGCTAACTTTTTTCTTTGTTATCTTTTCAG-<br>TTAATGTGGTTGCGAAGGAATTTACCTTAGACTTCTCGACTGCAAAGACGTATGTAGATTCGCT<br>GAATGTCATTCGCTCTGCAATAGGTACTCCATTACAGACTATTTCATCAGGAGGTAC-<br>GTCTTTACTGATGATTGATAGTGGCACAGGGGATAATTTGTTTGCAGTTGATGTCAGAGGGAT<br>AGATCCAGAGGAAGGGCGGTTTAATAATCTACGGCTTATTGTTGAACGAAATAATTTA-<br>TATGTGACAGGATTTGTTAACAGGACAAATAATGTTTTTTATCGCTTTGCTGATTTTTCACATGT<br>TACCTTTCCAGGTACAACAGCGGTTACATTGTCTGGTGACAGTAGCTATACCAC-<br>GTTACAGCGTGTTGCAGGGATCAGTCGTACGGGGATGCAGATAAATCGCCATTCGTTGACTAC<br>TTCTTATCTGGATTTAATGTCGCATAGTGGAACCTCACTGACGCAGTCTGTGGCAA-<br>GAGCGATGTTACGGTTTGTTACTGTGACAGCTGAAGCTTTACGTTTTCGGCAAATACAGAGGG<br>GATTTCGTACAACACTGGATGATCTCAGTGGGCGTTCTTATGTAATGACTGCTGAAGATGTT-<br>GATCTTACATTGAACTGGGGAAGGTTGAGTAGTGTCCTGCCTGATTATCATGGACAAGACTCT<br>GTTCGTGTAGGAAGAATTTCTTTTGGAAGCATTAATGCAATTCTGGGAAGCGTGGCATTAA-<br>TACTGAATTGTCATCATCATGCATCGCGAGTTGCCAGAATGGCATCTGATGAGTTTCCTTCTAT<br>GTGTCAGGCAGATGGAAGAGTCCGTGGGATTACGCACAATAAAATATTGTGGGAT-<br>TCATCCACTCTGGGGGCAATTCTGATGCGCAGAACTATTAGCAGTTGAGGGGGTAAAATGAAA<br>AAAACA
VT2	ATATCGACCCCTCTTGAACATATATCTCAGGGGACCACATCGGTGTCTGTTATTAAC-<br>CACACCCCACCGGGCAGTTATTTTGCTGTGGATATACGAGGGCTTGATGTCTATCAGGCGCGT<br>TTTGACCATCTTCGTCTGATTATTGAGCAAAATAATTTATATGTGGCCGGGTTCGTTAATAC-<br>GGCAACAAATACTTTCTACCGTTTTTCAGATTTTACACATATATCAGTGCCCGGTGTGACAACG<br>GTTTCCATGACAACGGACAGCAGTTATACCACTCTGCAACGTGTCGCAGCGCTGGAAC-<br>GTTCCGGAATGCAAATCAGTCGTCACTCACTGGTTTCATCATATCTGGCGTTAATGGAGTTCA<br>GTGGTAATACAATGACCAGAGATGCATCCAGAGCAGTTCTGCGTTTTGTCACTGTCACAG-<br>CAGAAGCCTTACGCTTCAGGCAGATACAGAGAGAATTTCGTCAGGCACTGTCTGAAACTGCTC

	CTGTGTATACGATGACGCCGGGAGACGTGGACCTCACTCTGAACTGGGGGCGAATCAG-CAATGTGCTTCCGGAGTATCGGGGAGAGGATGGTGTCAGAGTGGGGAGAATATCCTTTAATA ATATATCAGCGATACTGGGGACTGTGGCCGTTATACTGAATT-GCCATCATCAGGGGGCGCGTTCTGTTCGCGCCGTGAATGAAGAGAGTCAACCAGAATGTCAG ATAACTGGCGACAGGCCCGTTATAAAAATAAACAATACATTATGGGAAAGTAATACAGCTG-CAGCGTTTCTGAACAGAAAGTCACAGTTTTTATATACAACGGGTAAATAAAGGAGTTAAGCATG AAGAAGATGTTTATGGCGGTTTTATTTGCATTAGCTTCTGTTAATGCAATGGCGGCG-GATTGTGCTAAAGGTAAAATTGAGTTTTCCAAGTATAATGAGGATGACACATTTACAGTGAAGG TTGACGGGAAAGAATACTGGACCAGTCGC

3. Supporting data

3.1. DNA/LYO3 MAST ISOPLEX® VTEC-Accelerated stability study

3.1.1. Method

The accelerated stability study was conducted to determine if the MAST ISOPLEX® *VTEC* kit can achieve a minimum shelf life of 1 year at 2°C to 8°C or ambient temperature and to establish the product's optimal storage temperature. The acceptance criteria used in the accelerated stability study are defined in Table S2.

Three batches of MAST ISOPLEX® *VTEC* pellets were manufactured, then sealed in aluminum bags with two desiccant sachets and stored at a range of temperatures (4⁰C, 22⁰C, 37⁰C, or 50⁰C) in designated temperature-controlled incubators. Each temperature was monitored on a continuous basis by data loggers. Aluminum bags from each batch would be opened at selected intervals (weeks 0, 1, 2, 3, 4, 6, 8, 10, 12, and 13 post-manufacture) and analyzed with set concentrations of *vt1*/*vt2* positive control (PC) DNA (10 pg/μL, 1 pg/μL, or 100 fg/μL) and primers to test the assay performance. The experiment was performed using positive control DNA included in the kit, reconstituted as per IFU, and then diluted down accordingly (neat, 1:10, or 1:100). The tests were performed using lyophilized LAMP reaction pellets. The assay was run for 60 min at 63°C and the fluorescent outputs were monitored in TAMRA (IC) and Cy5 (*vt1*) and FAM (*vt2*) channels using the Applied Biosystems® 7500 Real-Time PCR System (Applied Biosystems, Waltham, MA, USA). Each sample was tested in duplicate and two negative control samples were included at every time point, for every batch. In total, at each time point, each parameter (*vt1, vt2,* or IC) was tested in six replicates.

To determine the 'anticipated' stability of the product from the accelerated studies, the accelerated aging protocol formula was applied.

$R = 2^A X$

whereby

R = real-time equivalent

X= actual time under accelerated conditions

A= increase in temperature over normal storage conditions /10

The shelf life of the product is initially estimated using accelerated studies and then confirmed via real-time stability studies.

Table S2. Specification of acceptance criteria for the MAST ISOPLEX® *VTEC* kit applied in the accelerated stability study.

***vt1*/*vt2* DNA concentration**	***vt1*/*vt2* DNA amplification times**	**IC DNA amplification times**
10 pg/μL	≤15 min	< 40 min
1 pg/μL	≤20 min	

100 fg/μL	≤30 min	
No Template Control (NTC)	>40 min	

3.1.2. Results and discussion

Data loggers showed that each temperature was consistent across the study, apart from sporadic out-of-range spikes in temperature, which correspond to the time period when data collection from the data logger took place and the data logger was removed from the incubator (refer to Figure S1).

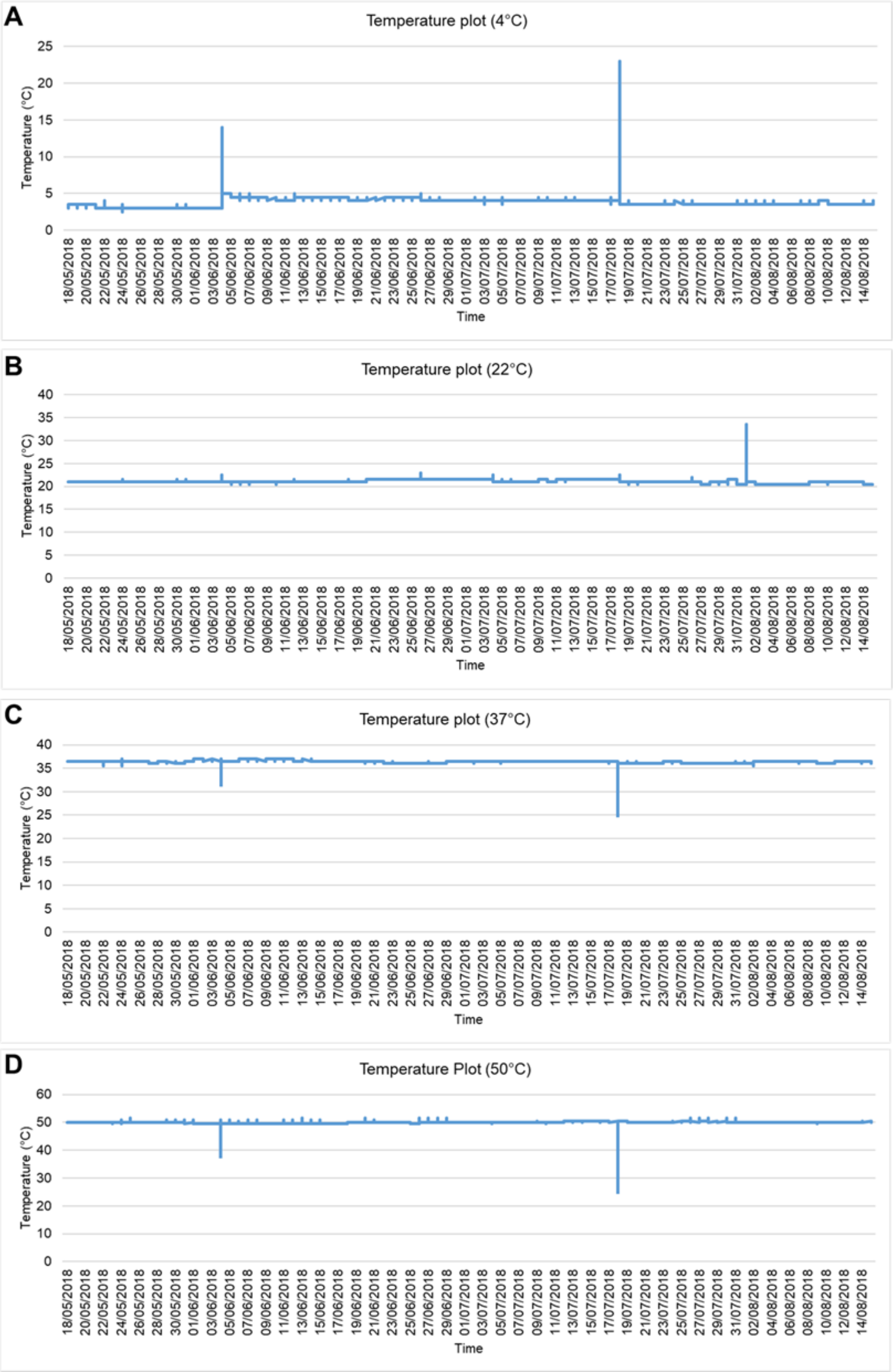

Figure S1. Temperature plots collected by data loggers were recorded throughout the duration of the MAST ISOPLEX® *VTEC* kit-accelerated stability study. A) Temperature plot at 4°; B) 22° Temperature plot; C) 37° Temperature plot; D) 50° Temperature plot.

The experiments demonstrated that all results (Ct values) for different concentrations of the positive control were within the specification (Figure S2 to Figure S5) for all primer

sets (*vt1, vt2,* and IC DNA). The Ct values for *vt1, vt2,* and IC obtained at the beginning of the study were comparable to those noted at week 13 even following storage at 50°C. These results demonstrate that the active components of the kit (BST polymerase, primers, and probes) remain fully functional even after exposure to temperatures as high as 50°C.

The analysis of no template control samples revealed that sporadic false amplification could be detected before the assay cut of point (40 min). To visualize the assay performance over time and to determine if there is a time-related trend between the prevalence of false positive results detected before the assay cut-off point and the time of exposure of the MAST ISOPLEX® *VTEC* kit to different temperatures, line charts were generated whereby the Ct values for each assay target were plotted against time (Figure S2 to Figure S5). A separate graph was generated for a dataset obtained at each temperature. The graphs did not demonstrate a clear correlation between the time and Ct values of occurring false positives for any of the temperatures tested.

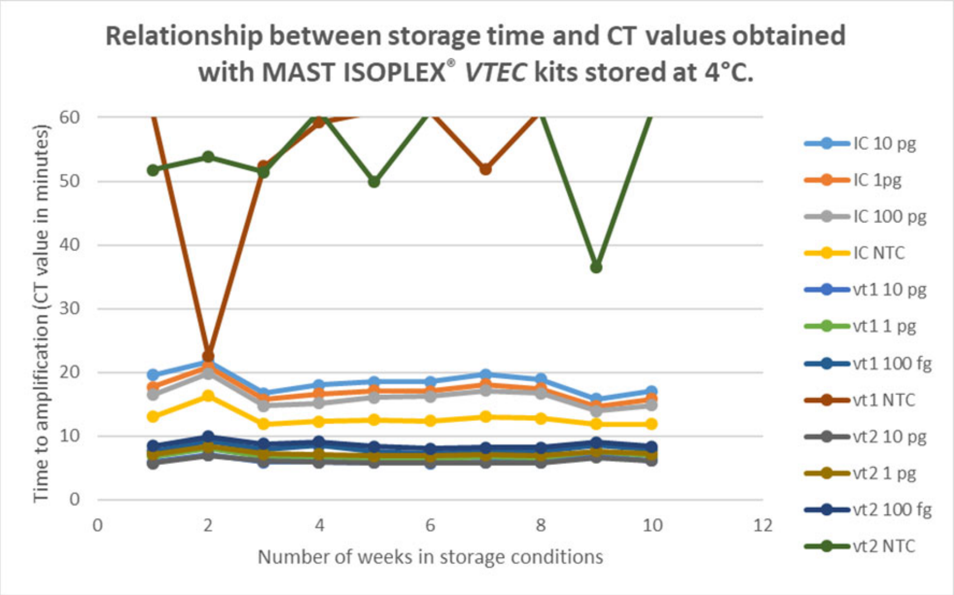

Figure S2. Relationship between storage time and Ct values obtained with MAST ISOPLEX® VTEC kits stored at 4°C. Depicted concentrations (10 pg, 1 pg, and 100 fg) refer to concentrations of vt1 and vt2 control DNA plasmids per reaction; the internal control plasmid DNA (IC) was used at 1 pg/ reaction in all samples including no template controls (NTC). Each data point represents the mean Ct value from six replicate tests.

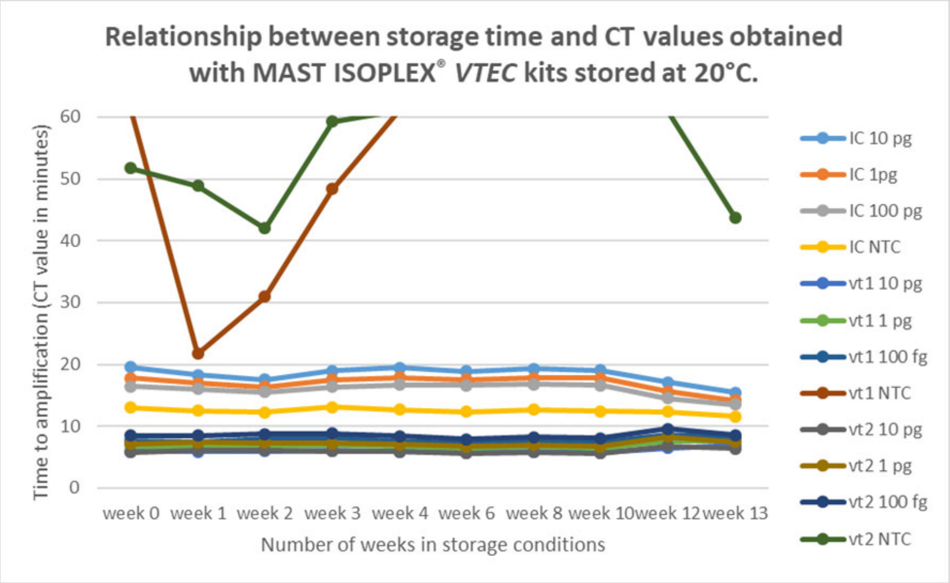

Figure S3. Relationship between storage time and Ct values obtained with MAST ISOPLEX® VTEC kits stored at 20°C. Depicted concentrations (10 pg, 1 pg, and 100 fg) refer to concentrations of vt1 and vt2 control DNA plasmids per reaction; the internal control plasmid DNA (IC) was used at 1 pg/ reaction in all samples including no template controls (NTC). Each data point represents the mean Ct value from six replicate tests.

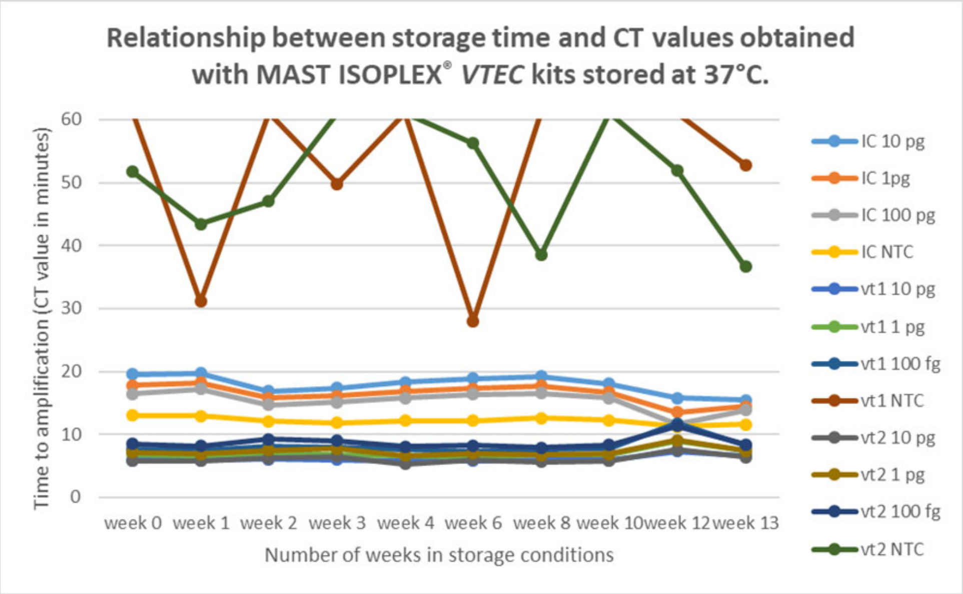

Figure S4. Relationship between storage time and Ct values obtained with MAST ISOPLEX® VTEC kits stored at 37°C. Depicted concentrations (10 pg, 1 pg, and 100 fg) refer to concentrations of vt1 and vt2 control DNA plasmids per reaction; the internal control plasmid DNA (IC) was used at 1 pg/ reaction in all samples including no template controls (NTC). Each data point represents the mean Ct value from six replicate tests.

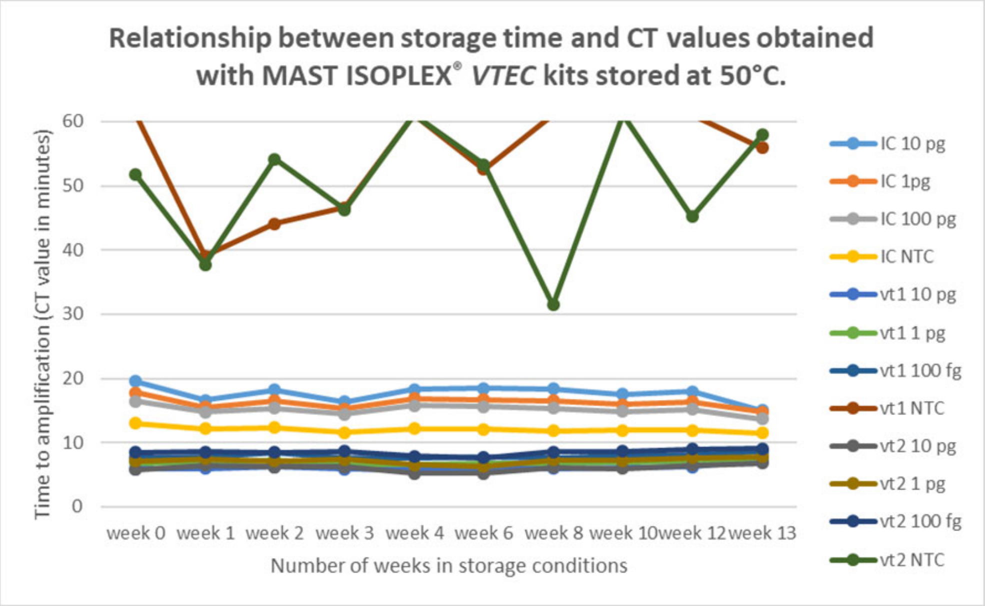

Figure S5. Relationship between storage time and Ct values obtained with MAST ISOPLEX® VTEC kits stored at 50°C. Depicted concentrations (10 pg, 1 pg, and 100 fg) refer to concentrations of vt1 and vt2 control DNA plasmids per reaction; the internal control plasmid DNA (IC) was used at 1 pg/ reaction in all samples including no template controls (NTC). Each data point represents the mean Ct value from six replicate tests.

In-depth analysis of the raw data revealed that the false application results before the assay cut-off point (40 min) occur sporadically in random replicates and can be observed at any of the tested temperatures. The number of false positive results in NTCs at each tested temperature is presented in Tables S3 to S6. The relationship between storage temperature and assay specificity is shown in Figure S6 and Figure S7.

Table S3. Number of false positive results generated before 40 min with the MAST ISOPLEX® *VTEC* kit stored at 4°C.

Week of storage at 4°C	Number of false positive results per 6 replicates in FAM channel	Number of false positive results per 6 replicates in CY5 channel
0	0	0
1	0	1
2	0	0
3	0	0
4	0	0
6	0	0
8	0	0
10	0	0
12	1	0
13	0	0

Table S4. Number of false positive results generated before 40 min with the MAST ISOPLEX® *VTEC* kit stored at 20°C.

Week of storage at 20°C	Number of false positive results per 6 replicates in FAM channel	Number of false positive results per 6 replicates in CY5 channel

0	0	0
1	0	1
2	0	2
3	0	0
4	0	0
6	0	0
8	0	0
10	0	0
12	0	0
13	0	0

Table S5. Number of false positive results generated before 40 min with the MAST ISOPLEX® *VTEC* kit stored at 37°C.

Week of storage at 37°C	Number of false positive results per 6 replicates in FAM channel	Number of false positive results per 6 replicates in CY5 channel
0	0	0
1	0	1
2	0	0
3	0	0
4	0	0
6	0	1
8	1	0
10	0	0
12	0	0
13	1	0

Table S6. Number of false positive results generated before 40 min with the MAST ISOPLEX® *VTEC* kit stored at 50°C.

Week of storage at 50°C	Number of false positive results per 6 replicates in FAM channel	Number of false positive results per 6 replicates in CY5 channel
0	0	0
1	1	2
2	0	0
3	1	1
4	0	0
6	0	0
8	1	0
10	1	0
12	0	0
13	0	0

A correlation between MAST ISOPELX® *VTEC* kit storage temperature and the number of false positive results in NTCs or the assay specificity is demonstrated in Figure S6 and Figure S7, respectively.

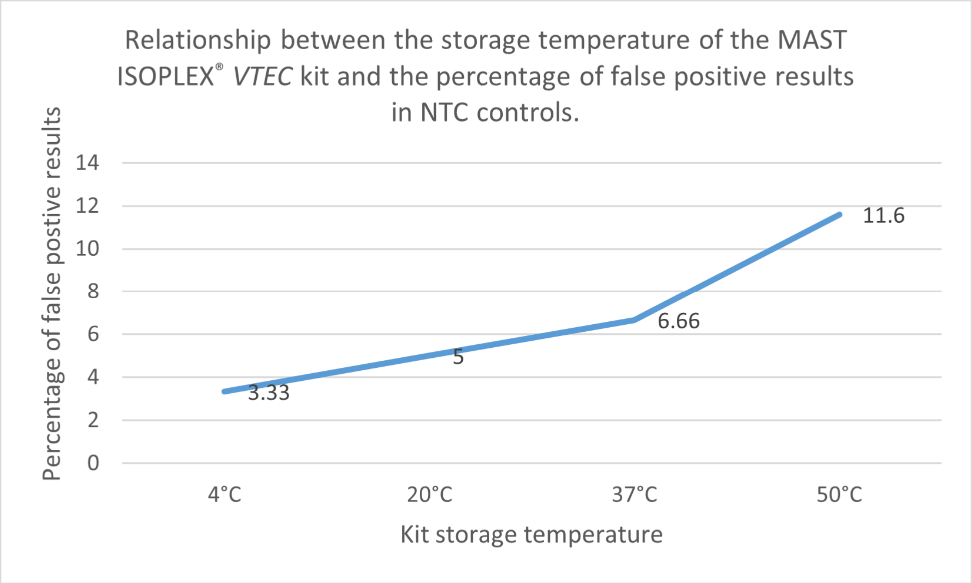

Figure S6. Relationship between the storage temperature of the MAST ISOPLEX® *VTEC* kit and the percentage of false positive results in NTC controls.

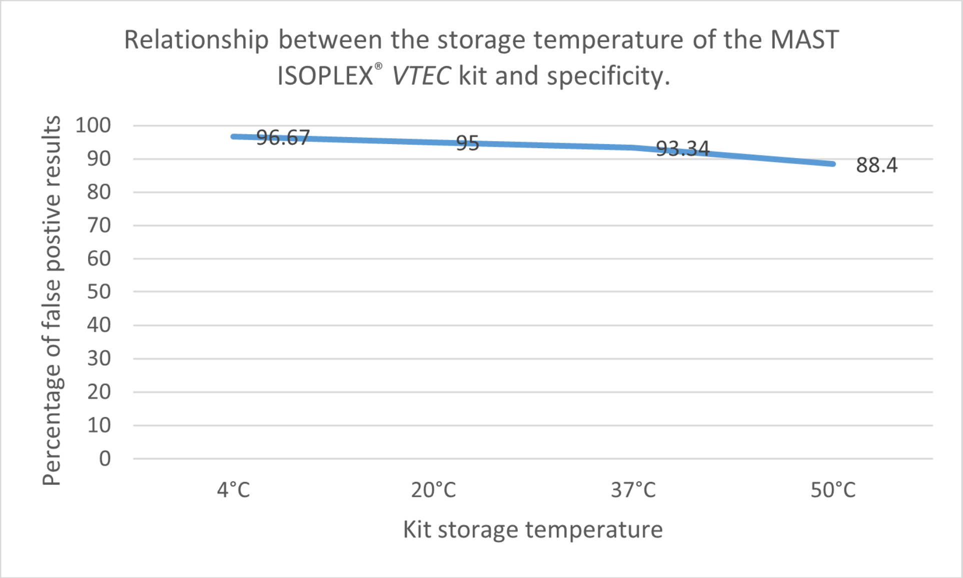

Figure S7. Relationship between the storage temperature of the MAST ISOPLEX® *VTEC* kit and specificity.

3.1.3. Conclusions

The analysis of results from the accelerated stability study suggests that the active ingredients present in the MAST ISOPLEX® *VTEC* kit maintain their activity for at least 13 weeks at 50°C, equating to a shelf life of 6.06 years at 4°C or 13 months at 30°C. The lowest percentage of false positive results was observed when the product was stored at 4°C (3.33%) and therefore a decision was made to conduct a real-time stability study at this temperature.

3.2. DNA/LYO3 MAST ISOPLEX® VTEC real-time stability study report

3.2.1. Method

A real-time stability study was conducted on MAST ISOPLEX® *VTEC* product lot number: 413510. Each kit was stored unopened, at the recommended storage temperature of 2–8°C for the duration of the shelf life. The product was assessed at 0, 4, 5, 6, 12, 18, and 24 months post manufacturing. At each time point, positive control DNA at 10 pg/µl (*vt1 vt2* DNA) included in the kit was tested in triplicate alongside a negative control where molecular grade water from the kit replaced the DNA sample. The assay was set up as per IFU and was run for 60 min at 63°C with FAM/TAMRA/Cy5 channels using the Applied Biosystems® 7500 Real-Time PCR Systems instrument (Applied Biosystems, Waltham, MA, USA). The experiment demonstrated that results for all primer sets (*vt1, vt2,* and IC DNA) were within the specification shown in Table S7. The results have been summarized in Table S8.

Table S7. Specification of acceptance criteria for the real-time stability study of the MAST ISOPLEX® *VTEC* kit.

***vt1/vt2* DNA concentration**	***vt1/vt2* DNA amplification times**	**IC DNA amplification times**
10 pg/µl	≤15 min	<40 min
No Template Control (NTC)	>40 min	

3.2.2. Results

Table S8. Ct values obtained with the MAST ISOPLEX® *VTEC* kit during the real-time stability study at 4°C.

**MAST ISOPLEX® *VTEC* pellets stored at 4⁰C. Mean Ct values of each sample +/- standard deviation are shown. NTC = No Template Control; PC = Positive control DNA (*vt1* or *vt2* control plasmid); ND = not determined, the target did not amplify.**		
**Time zero**		
**Probe type**	10 pg PC DNA	NTC
vt1-CY5	9.46 ± 0.19	ND
vt2-FAM	11.09 ± 0.62	ND
IC-TAMRA	18.24 ± 1.83	14.37 ± 0.64
Month 4		
Probe type	10 pg PC DNA	NTC
vt1-CY5	9.36 ± 0.10	ND
vt2-FAM	10.85 ± 0.12	ND
IC-TAMRA	17.79 ± 0.17	14.85 ± 0.02
Month 5		
Probe type	10 pg PC DNA	NTC
vt1-CY5	9.03 ± 0.06	ND
vt2-FAM	10.20 ± 0.28	ND
IC-TAMRA	18.82 ± 0.12	15.67 ± 0.07
Month 6		
Probe type	10 pg PC DNA	NTC
vt1-CY5	8.96 ± 0.22	ND
vt2-FAM	10.74 ± 0.25	ND
IC-TAMRA	16.86 ± 0.19	14.46 ± 0.06
Month 12		
Probe type	10 pg PC DNA	NTC

vt1-CY5	9.11 ± 0.12	ND
vt2-FAM	10.21 ± 0.17	ND
IC-TAMRA	19.49 ± 0.17	15.13 ± 0.21
Month 18		
Probe type	10 pg PC DNA	NTC
vt1-CY5	9.28 ± 0.07	ND
vt2-FAM	10.48 ± 0.14	ND
IC-TAMRA	16.48 ± 0.24	13.65 ± 0.12
Month 24		
Probe type	10 pg PC DNA	NTC
vt1-CY5	8.70 ± 0.03	ND
vt2-FAM	10.68 ± 0.29	ND
IC-TAMRA	15.21 ± 0.26	12.70 ± 0.14

3.2.3. Conclusions

The experiments from the first product lot# 413510 demonstrated that results for all primer sets (*vt1, vt2,* and IC DNA) were within the specification, suggesting that MAST ISOPLEX® *VTEC* is stable for at least 2 years at the recommended storage condition of 2°C to 8°C.

3.3. DNA/LYO3 MAST ISOPLEX® VTEC in-use stability study

3.3.1. Method

In-use stability data were generated on one batch of MAST ISOPLEX® *VTEC* (product lot number: 413510). The kit was stored at 2°C to 8°C unopened and away from the direct sunlight. After storage for 4 months, all reagents were reconstituted and then placed at −20°C. The frozen reagents were defrosted and tested again at the following time points: 5, 6, 12, 18, or 24 months. Tests were performed using positive control DNA reconstituted and stored at −20°C or water as a negative control, as per IFU. The assay was run for 40 min at 63°C with FAM/TAMRA/CY5 channels using the Applied Biosystems® 7500 Real-Time PCR Systems instrument (Applied Biosystems, Madison, WI, USA). Each sample was tested in triplicate and three negative control samples were included at every time point.

3.3.2. Results

The experiment reported that results for all primer sets (*vt1, vt2,* and IC DNA) were within the specification shown in Table S9. The results are summarized in Table S10.

Table S9. Specification of acceptance criteria for the in-use stability study of the MAST ISOPLEX® *VTEC* kit.

***vt1/vt2* DNA concentration**	***vt1/vt2* DNA amplification times**	**IC DNA amplification times**
10 pg/µl	≤ 15 min	< 40 min
No Template Control (NTC)	> 40 min	

Table S10. Results of the in-use stability study of the MAST ISOPLEX® *VTEC* kit.

**MAST ISOPLEX® *VTEC* reconstituted lyophilized component stored at −20⁰C. Mean Ct values of each sample ± standard deviation are shown. NTC = No Template**

**Control; PC = Positive control DNA (*vt1* or *vt2* control plasmid); ND = not determined, the target did not amplify.**		
**Time zero**		
**Probe type**	10 pg PC DNA	NTC
vt1-CY5	9.46 ± 0.19	ND
vt2-FAM	11.09 ± 0.62	ND
IC-TAMRA	18.24 ± 1.83	14.37 ± 0.64
Month 5		
Probe type	10 pg PC DNA	NTC
vt1-CY5	9.20 ± 0.15	ND
vt2-FAM	10.09 ± 0.08	ND
IC-TAMRA	18.23 ± 0.26	15.05 ± 0.03
Month 6		
Probe type	10 pg PC DNA	NTC
vt1-CY5	8.91 ± 0.29	ND
vt2-FAM	10.58 ± 0.17	ND
IC-TAMRA	16.66 ± 0.22	15.16 ± 1.74
Month 12		
Probe type	10 pg PC DNA	NTC
vt1-CY5	8.56 ± 0.46	ND
vt2-FAM	9.91 ± 0.12	ND
IC-TAMRA	16.22 ± 0.51	13.41 ± 0.49
Month 18		
Probe type	10 pg PC DNA	NTC
vt1-CY5	9.45 ± 0.27	ND
vt2-FAM	10.59 ± 0.28	ND
IC-TAMRA	16.28 ± 0.24	13.64 ± 0.06
Month 24		
Probe type	10 pg PC DNA	NTC
vt1-CY5	9.05 ± 0.10	ND
vt2-FAM	10.94 ± 0.11	ND
IC-TAMRA	15.97 ± 0.38	13.36 ± 0.18

3.3.3. Conclusions

The study demonstrated that results for all primer sets (*vt1, vt2,* and IC DNA) were within the specification, and therefore lyophilized components reconstituted in the appropriate buffer, as per IFU, are stable after 2 years of storage at −20°C.

3.4. DNA/LYO3 MAST ISOPLEX®VTEC-simulated shipping stability study

3.4.1. Simulated shipping study—Overview

The simulated shipping stability study was performed using simulated conditions taking into account variations in temperature and humidity that may occur during product transit. The simulated shipping model used in the study was set up based on World Health Organization WHO recommendations and data collected from temperature/ humidity data loggers during the shipment of MAST products in real-life scenarios (Figure S8). This model accounts for the longest transit time and highest temperature and humidity seen within the real-time shipping study.

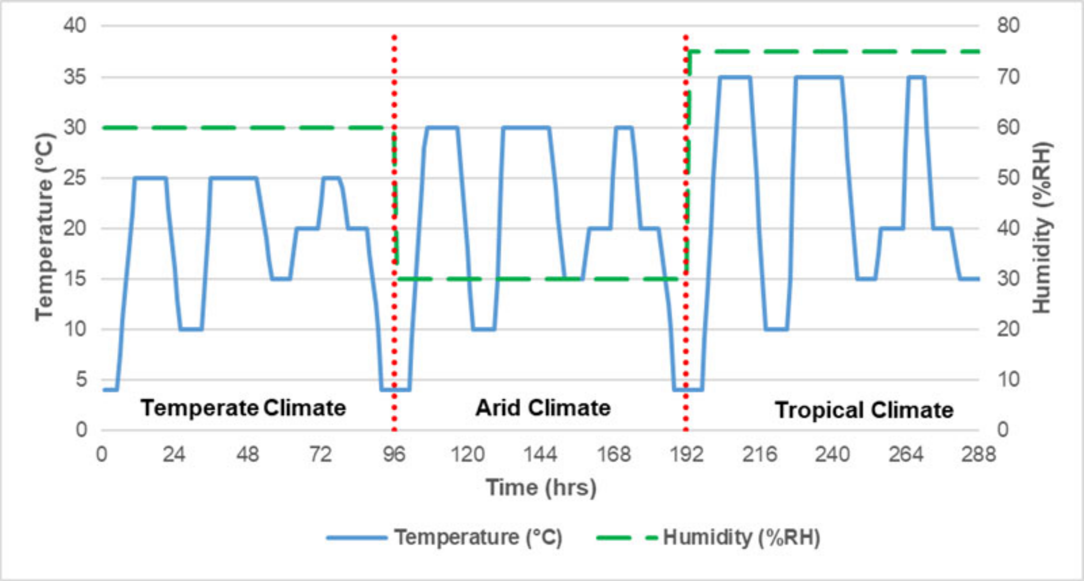

Figure S8. Worst-case-scenario temperature and humidity plot for simulated shipping stability.

3.4.2. Method

Three DNA/LYO3 MAST ISOPLEX® *VTEC* kits from the same batch (lot 060723) were used in this study, labeled as shipping stability time zero (Ts0), time 1 (Ts1), and "control" (Ts Control). Prior to exposing one kit to the simulated shipping stability cycle (Ts1), another kit was tested on the same day (Ts0). The third kit was kept at the recommended storage conditions of 4°C throughout the entire duration of the study (Ts control) and tested alongside Ts1 upon 288 hours of cycle completion (Table S11). The Ts1 kit was packed into a shipping box, sealed with Mast shipping tape, and placed into a temperature cycling climate chamber, where the simulated shipping program "JWALL" was run (Table S12). Upon cycle completion, the box was unpacked and the Ts1 kit was opened for testing. Each kit was tested with positive control DNA at 10 pg/µL (*vt1 vt2* DNA) included in the kit in triplicate alongside a negative control where molecular grade water from the kit replaced the DNA sample. The assay was set up as per IFU and was run for 40 min at 63°C with FAM/TAMRA/Cy5 channels using the ABI7500 instrument. The specifications of the acceptance criteria are shown in Table S13.

Table S11. Simulated shipping stability study testing schedule.

Kit(s) tested	Time point	Date of testing
Ts0	zero	24-Aug–23
Ts1, Ts control	288 h	5-Sep–23

h

Table S12. Shipping simulation cycle details.

Program Name: JWALL				
**Temperature setting**	**Ramp Number**	**Duration (hh:mm)**	**Set Temperature (°C)**	**Humidity (%RH)**
Constant Temperature	01/02	05:00	4	60
Ramp Temperature	03/02	06:00	4–25	60
Constant Temperature	04/02	10:00	25	60
Ramp Temperature	05/02	05:00	25–10	60
Constant Temperature	06/02	07:00	10	60
Ramp Temperature	07/02	03:00	10–25	60

Program Name: JWALL				
**Temperature setting**	**Ramp Number**	**Duration (hh:mm)**	**Set Temperature (°C)**	**Humidity (%RH)**
Constant Temperature	08/02	15:00	25	60
Ramp Temperature	09/02	05:00	25–15	60
Constant Temperature	10/02	06:00	15	60
Ramp Temperature	11/02	02:00	15–20	60
Constant Temperature	12/02	07:00	20	60
Ramp Temperature	13/02	02:00	20–25	60
Constant Temperature	14/02	05:00	25	60
Ramp Temperature	15/02	03:00	25–20	60
Constant Temperature	16/02	06:00	20	60
Ramp Temperature	17/02	06:00	20–4	60
Constant Temperature	18/19	08:00	4	60–30
Ramp Temperature	21/20	06:00	4–30	30
Constant Temperature	22/20	10:00	30	30
Ramp Temperature	23/20	05:00	30–10	30
Constant Temperature	24/20	07:00	10	30
Ramp Temperature	25/20	03:00	10–30	30
Constant Temperature	26/20	15:00	30	30
Ramp Temperature	27/20	05:00	30–15	30
Constant Temperature	28/20	06:00	15	30
Ramp Temperature	29/20	02:00	15–20	30
Constant Temperature	30/20	07:00	20	30
Ramp Temperature	31/20	02:00	20–30	30
Constant Temperature	32/20	05:00	30	30
Ramp Temperature	33/20	03:00	30–20	30
Constant Temperature	34/20	06:00	20	30
Ramp Temperature	35/20	06:00	20–4	30
Constant Temperature	36/37	08:00	4	30–75
Ramp Temperature	40/38	06:00	4–35	75
Constant Temperature	41/38	10:00	35	75
Ramp Temperature	42/38	05:00	35–10	75
Constant Temperature	43/38	07:00	10	75
Ramp Temperature	44/38	03:00	10–35	75
Constant Temperature	45/38	15:00	35	75
Ramp Temperature	46/38	05:00	35–15	75
Constant Temperature	47/38	06:00	15	75
Ramp Temperature	48/38	02:00	15–20	75
Constant Temperature	49/38	07:00	20	75
Ramp Temperature	50/38	02:00	20–35	75
Constant Temperature	51/38	05:00	35	75
Ramp Temperature	52/38	03:00	35–20	75
Constant Temperature	53/38	06:00	20	75
Ramp Temperature	54/38	03:00	20–15	75
Constant Temperature	55/38	06:00	15	75

Table S13. Specification of the acceptance criteria for the MAST ISOPLEX® *VTEC* kit applied in the simulated shipping study.

***vt1/vt1* DNA concentration**	***vt1/vt1* DNA amplification times**	**IC DNA amplification times**
10 pg/µl	≤ 15 min	< 40 min
No Template Control (NTC)	> 40 min	

3.4.3. Simulated shipping study—Results

Results for all primer sets (VT1, VT2, and IC DNA) were the specification at all conditions tested (Table S14).

Table S14. Results of the simulated shipping stability study of the MAST ISOPLEX® *VTEC* kit.

**MAST ISOPLEX® *VTEC* simulated shipping stability study. Mean Ct values of each sample ± standard deviation are shown. NTC = No Template Control; PC = Positive control DNA (*vt1* or *vt2* control plasmid); ND = not determined, the target did not amplify.**						
	**Ts0**		**Ts control**		**Ts1**	
**Probe type**	**10 pg PC DNA**	NTC	10 pg PC DNA	NTC	10 pg PC DNA	NTC
vt1-CT5	8.19 ±0.49	ND	6.55 ±0.08	53.08	6.56 ±0.05	ND
vt2-FAM	10.87 ±0.74	ND	6.60 ±0.05	ND	6.32 ±0.08	ND
IC-TAMRA	29.32 ±0.33	18.66 ±1.85	31.23 ±0.48	20.18 ±0.03	30.69 ±0.63	19.76 ±0.28

3.4.4. Conclusions

The simulated shipping conditions tested have shown that the product is not impacted by the current shipping conditions. Results for the simulated shipping kits are within the acceptable criteria; therefore, MAST ISOPLEX® *VTEC* can be shipped at room temperature.

The data collected during real-time and accelerated stability studies demonstrate that the product is stable for at least 2 years at 2°C to 8°C. These storage conditions (2°C to 8°C) were chosen based on the analysis of the incidence of false positive results occurring before the assay cut-off point (40 min), which was lowest when the product was stored at 2°C to 8°C.

3.5. Supplementary raw data

Table S15. Raw data generated with the ABI7500 instrument used to generate Figure 3.

**ABI7500**					
***vt1*-CY5**					
**lot number**	**409288**	**409315**	**409322**	mean	SD
Positive control	8.84	8.70	9.22	8.92	0.27
NTCs	ND	55.25	59.33	ND	ND
vt2-FAM					
lot number	409288	409315	409322	mean	SD
Positive control	8.25	8.77	8.68	8.57	0.28
NTCs	ND	56.04	ND	ND	ND
IC TAMRA					
lot number	409288	409315	409322	mean	SD

pos	14.57	13.01	10.08	12.55	2.28
NTCs	11.38	11.20	9.37	10.65	1.11

Table S16. Raw data generated with the Mic instrument used to generate Figure 3.

**MIC**					
***vt1*-CY5**					
**lot 0120233 n1**	**lot 0120233 n2**	**lot 0120233 n3**	**lot 0120233 n4**	mean	SD
5.58	5.43	5.54	5.52	5.52	0.06
34	ND	ND	36	ND	ND
vt2-FAM					
n1	n2	n3	n4	mean	SD
7.81	7.58	7.25	7.62	7.57	0.23
ND	ND	ND	ND	ND	ND
IC TAMRA					
n1	n2	n3	n4	mean	SD
5.38	5.44	5.42	5.60	5.46	0.10
5.28	5.41	5.24	5.41	5.34	0.09

Table S17. Statistical analysis of data is presented in Figure 3.

**Statistical analysis**		
**Target**	**t test (ABI vs Mic) *p* value**	delta mean Ct
vt1-CY5 Positive control	1.85 E−06	3.40
vt2-FAM Positive control	0.003442	1.00
IC TAMRA Positive control	0.001348	7.09
IC TAMRA NTCs	0.000184	5.32

Table S18. Raw data used to perform Probit analysis and determine the LOD of the *vt1*-CY assay with Minitab® Statistical Software v18 (Minitab®, LLC.).

***vt1* plasmid**				
**Plasmid fg/ul**	**No. copies**	**Log. No. copies**	**No. replicates**	No. positives
0.5 fg/ul	135.45	2.131779009	9	9
0.1 fg/ul	27.09	1.432809005	9	8
0.005 fg/ul	1.3545	0.131779009	9	1
0.001 fg/ul	0.2709	−0.567190995	9	2
0.00005 fg/ul	0.013545	−1.868220991	9	0
Table of Percentiles				
		Standard	95.0% Fiducial CI	
Percent	Percentile	Error	Lower	Upper
1	−1.39246	0.494319	−3.14305	−0.699979
2	−1.16902	0.446799	−2.73096	−0.534947
3	−1.02726	0.417488	−2.47129	−0.428444
4	−0.920612	0.395974	−2.27711	−0.347165
5	−0.833864	0.378874	−2.12004	−0.280172
6	−0.760029	0.364643	−1.98707	−0.222433
7	−0.69529	0.35244	−1.87109	−0.17119

8	−0.637323	0.341755	−1.7678	−0.12476
9	−0.584605	0.332256	−1.67436	−0.0820356
10	−0.536078	0.323711	−1.5888	−0.0422472
20	−0.175482	0.267964	−0.971623	0.271967
30	0.0845326	0.239795	−0.557155	0.5291
40	0.306706	0.2271	−0.234058	0.779862
50	0.514366	0.226446	0.0360047	1.04617
60	0.722026	0.236745	0.274789	1.34376
70	0.944199	0.258385	0.500953	1.69145
80	1.20421	0.294394	0.737844	2.12616
90	1.56481	0.356961	1.03575	2.75966
91	1.61334	0.366158	1.07404	2.8467
92	1.66605	0.376311	1.11529	2.94163
93	1.72402	0.387655	1.16024	3.0464
94	1.78876	0.400527	1.20999	3.16386
95	1.8626	0.415444	1.26621	3.29835
96	1.94934	0.433258	1.33165	3.45698
97	2.05599	0.455534	1.41128	3.65281
98	2.19775	0.485694	1.51596	3.91429
99	2.42119	0.534261	1.67878	4.3286
	Percentile	Error	Lower Limit	Upper Limit
DNA (*vt1* plasmid) copies per reaction	72.88	2.60	18.46	1987.70

Table S19. Raw data used to perform Probit analysis and determine the LOD of the *vt2*-FAM assay with Minitab® Statistical Software v18 (Minitab®, LLC.).

***vt2* plasmid**				
**Plasmid fg/ul**	**No. copies**	**Log. No. copies**	**No. replicates**	No. positives
0.5 fg/ul	134.7	2.129367596	9	9
0.1 fg/ul	26.94	1.430397591	9	9
0.005 fg/ul	1.347	0.129367596	9	2
0.001 fg/ul	0.2694	−0.569602409	9	1
0.00005 fg/ul	0.01347	−1.870632404	9	0
Table of Percentiles				
		Standard	95.0% Fiducial CI	
Percent	Percentile	Error	Lower	Upper
1	−0.956105	0.37768	−2.62134	−0.451223
2	−0.802581	0.33846	−2.26163	−0.339816
3	−0.705175	0.314564	−2.03571	−0.266837
4	−0.631901	0.29723	−1.86729	−0.2104
5	−0.572297	0.283615	−1.73149	−0.1633
6	−0.521566	0.272422	−1.61689	−0.122213
7	−0.477084	0.262946	−1.51729	−0.0853152
8	−0.437256	0.25476	−1.4289	−0.0514893
9	−0.401033	0.247585	−1.34924	−0.0199992
10	−0.367691	0.241227	−1.27659	0.0096689
20	−0.119929	0.2035	−0.765048	0.258431
30	0.0587252	0.190316	−0.44279	0.484403
40	0.211378	0.190705	−0.210014	0.720069

50	0.35406	0.200816	−0.0281131	0.976009
60	0.496741	0.219037	0.126082	1.25966
70	0.649394	0.245457	0.26965	1.58453
80	0.828048	0.282802	0.419773	1.98264
90	1.07581	0.341926	0.609411	2.5533
91	1.10915	0.350334	0.633859	2.63117
92	1.14538	0.359563	0.660199	2.71598
93	1.1852	0.369816	0.688921	2.80948
94	1.22968	0.381387	0.720728	2.91417
95	1.28042	0.394725	0.756689	3.03389
96	1.34002	0.410567	0.798555	3.17493
97	1.41329	0.43027	0.849526	3.34881
98	1.5107	0.456795	0.916557	3.58069
99	1.66422	0.499239	1.02084	3.94752
	Percentile	Error	Lower limit	Upper limit
DNA copies (*vt2* plasmid) per reaction	19.07	2.48	5.71	1081.16

Table S20. Raw data obtained with the IC-TAMRA probe during accelerated stability studies (product stored at 4°C).

**4°C IC-TAMRA probe**				
**Week**	**Target**	**Plasmid concentration per reaction**	**mean Ct n = 3**	SD
week 0	IC-TAMRA	10 pg	19.5587	1.7049
week 0		1 pg	17.7458	1.3762
week 0		100 fg	16.4827	1.1691
week 0		NTC	13.0085	0.7622
Week	Target	Plasmid concentration per reaction	mean Ct n = 3	SD
week 1	IC-TAMRA	10 pg	21.7597	7.3532
week 1		1 pg	20.9199	7.4718
week 1		100 fg	19.8759	7.1969
week 1		NTC	16.3038	6.3486
Week	Target	Plasmid concentration per reaction	mean Ct n = 3	SD
week 2	IC-TAMRA	10 pg	16.6934	0.8794
week 2		1 pg	15.7318	0.8840
week 2		100 fg	14.7519	0.8144
week 2		NTC	11.8706	0.3492
Week	Target	Plasmid concentration per reaction	mean Ct n = 3	SD
week 3	IC-TAMRA	10 pg	18.0463	1.2545
week 3		1 pg	16.6737	1.0675
week 3		100 fg	15.1853	1.0319
week 3		NTC	12.2801	0.4849
Week	Target	Plasmid concentration per reaction	mean Ct n = 3	SD
week 4	IC-TAMRA	10 pg	18.4928	1.3626
week 4		1 pg	17.1185	1.2781

Week	Target	Plasmid concentration per reaction	mean Ct n = 3	SD
week 4		100 fg	16.0590	1.2434
week 4		NTC	12.5381	0.6530
Week	Target	Plasmid concentration per reaction	mean Ct n = 3	SD
week 6	IC-TAMRA	10 pg	18.5276	1.2257
week 6		1 pg	17.0916	1.1382
week 6		100 fg	16.2423	0.9091
week 6		NTC	12.3672	0.4982
Week	Target	Plasmid concentration per reaction	mean Ct n = 3	SD
week 8	IC-TAMRA	10 pg	19.6617	1.3556
week 8		1 pg	18.1365	1.1185
week 8		100 fg	17.1734	1.1496
week 8		NTC	13.0818	0.5733
Week	Target	Plasmid concentration per reaction	mean Ct n = 3	SD
week 10	IC-TAMRA	10 pg	18.9449	1.1622
week 10		1 pg	17.5040	1.1680
week 10		100 fg	16.7482	1.1801
week 10		NTC	12.7722	0.4946
Week	Target	Plasmid concentration per reaction	mean Ct n = 3	SD
week 12	IC-TAMRA	10 pg	15.8408	0.6053
week 12		1 pg	14.6491	0.5131
week 12		100 fg	13.9590	0.4965
week 12		NTC	11.9283	0.1131
Week	Target	Plasmid concentration per reaction	mean Ct n = 3	SD
week 13	IC-TAMRA	10 pg	17.0519	0.3848
week 13		1 pg	15.8045	0.2482
week 13		100 fg	14.8463	0.2111
week 13		NTC	11.9156	0.1086

Table S21. Raw data obtained with the *vt1*-CY5 probe during accelerated stability studies (product stored at 4°C).

**4°C *vt1*-CY5 probe**				
**Week**	**Target**	**Plasmid concentration per reaction**	**mean Ct n = 3**	SD
week 0	*vt1*-CY5	10 pg	5.8972	0.1624
week 0		1 pg	6.8234	0.2032
week 0		100 fg	7.6503	0.2367
week 0		NTC	ND	N/A
Week	Target	Plasmid concentration per reaction	mean Ct n = 3	SD
week 1	*vt1*-CY5	10 pg	7.0956	1.8598
week 1		1 pg	8.0661	2.2593
week 1		100 fg	9.2865	2.4080
week 1		NTC	22.5246	N/A

Week	Target	Plasmid concentration per reaction	mean Ct n = 3	SD
week 2	*vt1*-CY5	10 pg	5.9389	0.0567
week 2		1 pg	6.8250	0.0739
week 2		100 fg	7.9275	0.1018
week 2		NTC	52.3047	N/A
Week	Target	Plasmid concentration per reaction	mean Ct n = 3	SD
week 3	*vt1*-CY5	10 pg	5.9452	0.0922
week 3		1 pg	6.8332	0.0972
week 3		100 fg	8.5729	0.2181
week 3		NTC	59.2342	N/A
Week	Target	Plasmid concentration per reaction	mean Ct n = 3	SD
week 4	*vt1*-CY5	10 pg	5.8235	0.0539
week 4		1 pg	6.5330	0.0977
week 4		100 fg	7.6155	0.1124
week 4		NTC	ND	N/A
Week	Target	Plasmid concentration per reaction	mean Ct n = 3	SD
week 6	*vt1*-CY5	10 pg	5.7231	0.0761
week 6		1 pg	6.4176	0.0841
week 6		100 fg	7.2659	0.1030
week 6		NTC	ND	N/A
Week	Target	Plasmid concentration per reaction	mean Ct n = 3	SD
week 8	*vt1*-CY5	10 pg	5.9077	0.0789
week 8		1 pg	6.7891	0.1468
week 8		100 fg	7.7229	0.1383
week 8		NTC	51.8553	N/A
Week	Target	Plasmid concentration per reaction	mean Ct n = 3	SD
week 10	*vt1*-CY5	10 pg	5.9126	0.1337
week 10		1 pg	6.6751	0.2254
week 10		100 fg	7.6813	0.1520
week 10		NTC	ND	N/A
Week	Target	Plasmid concentration per reaction	mean Ct n = 3	SD
week 12	*vt1*-CY5	10 pg	6.9409	0.2675
week 12		1 pg	7.5476	0.1757
week 12		100 fg	8.5072	0.1817
week 12		NTC	ND	N/A
Week	Target	Plasmid concentration per reaction	mean Ct n = 3	SD
week 13	*vt1*-CY5	10 pg	6.1733	0.1384
week 13		1 pg	6.8647	0.0802
week 13		100 fg	7.8533	0.1483
week 13		NTC	ND	N/A

Table S22. Raw data obtained with the *vt2*-FAM probe during accelerated stability studies (product stored at 4°C).

**4°C *vt2*-FAM probe**				
**Week**	**Target**	**Plasmid concentration per reaction**	**mean Ct n = 3**	SD
week 0	*vt2*-FAM	10 pg	5.7833	0.1700
week 0		1 pg	7.1467	0.1971
week 0		100 fg	8.4642	0.3362
week 0		NTC	51.7425	N/A
Week	Target	Plasmid concentration per reaction	mean Ct n = 3	SD
week 1	*vt2*-FAM	10 pg	7.0074	1.2534
week 1		1 pg	8.4155	1.6365
week 1		100 fg	9.9148	1.7564
week 1		NTC	53.8242	1.6977
Week	Target	Plasmid concentration per reaction	mean Ct n = 3	SD
week 2	*vt2*-FAM	10 pg	6.0462	0.2934
week 2		1 pg	7.2701	0.2529
week 2		100 fg	8.7775	0.2618
week 2		NTC	51.4195	8.9361
Week	Target	Plasmid concentration per reaction	mean Ct n = 3	SD
week 3	*vt2*-FAM	10 pg	5.8983	0.1271
week 3		1 pg	7.1137	0.1540
week 3		100 fg	9.1272	0.1516
week 3		NTC	ND	N/A
Week	Target	Plasmid concentration per reaction	mean Ct n = 3	SD
week 4	*vt2*-FAM	10 pg	5.8560	0.2260
week 4		1 pg	6.9213	0.2830
week 4		100 fg	8.4100	0.4229
week 4		NTC	49.8529	N/A
Week	Target	Plasmid concentration per reaction	mean Ct n = 3	SD
week 6	*vt2*-FAM	10 pg	5.8143	0.1661
week 6		1 pg	6.9222	0.1452
week 6		100 fg	8.0742	0.2055
week 6		NTC	ND	N/A
Week	Target	Plasmid concentration per reaction	mean Ct n = 3	SD
week 8	*vt2*-FAM	10 pg	5.8304	0.1274
week 8		1 pg	7.0637	0.1776
week 8		100 fg	8.2057	0.1741
week 8		NTC	ND	N/A
Week	Target	Plasmid concentration per reaction	mean Ct n = 3	SD
week 10	*vt2*-FAM	10 pg	5.8719	0.1468
week 10		1 pg	6.9692	0.1633

Week	Target	Plasmid concentration per reaction	mean Ct n = 3	SD
week 10		100 fg	8.2509	0.2261
week 10		NTC	61.0000	#DIV/0!
Week	Target	Plasmid concentration per reaction	mean Ct n = 3	SD
week 12	*vt2*-FAM	10 pg	6.6770	0.3575
week 12		1 pg	7.6727	0.2063
week 12		100 fg	9.0385	0.3744
week 12		NTC	36.4400	8.2735
Week	Target	Plasmid concentration per reaction	mean Ct n = 3	SD
week 13	*vt2*-FAM	10 pg	6.2063	0.1160
week 13		1 pg	7.1978	0.1152
week 13		100 fg	8.3995	0.2312
week 13		NTC	ND	N/A

Table S23. Raw data obtained with the IC-TAMRA probe during accelerated stability studies (product stored at 20°C).

**20°C IC-TAMRA probe**				
**Week**	**Target**	**Plasmid concentration per reaction**	**mean Ct n = 3**	SD
week 0	IC-TAMRA	10 pg	19.5587	1.7049
week 0		1 pg	17.7458	1.3762
week 0		100 fg	16.4827	1.1691
week 0		NTC	13.0085	0.7622
Week	Target	Plasmid concentration per reaction	mean Ct n = 3	SD
week 1	IC-TAMRA	10 pg	18.2613	1.3138
week 1		1 pg	16.9267	1.6079
week 1		100 fg	16.0216	0.8466
week 1		NTC	12.5207	0.4801
Week	Target	Plasmid concentration per reaction	mean Ct n = 3	SD
week 2	IC-TAMRA	10 pg	17.5174	0.8248
week 2		1 pg	16.3533	0.8636
week 2		100 fg	15.5305	1.0008
week 2		NTC	12.2219	0.6773
Week	Target	Plasmid concentration per reaction	mean Ct n = 3	SD
week 3	IC-TAMRA	10 pg	18.9710	1.0967
week 3		1 pg	17.5471	1.2010
week 3		100 fg	16.3459	0.7158
week 3		NTC	13.0503	0.8264
Week	Target	Plasmid concentration per reaction	mean Ct n = 3	SD
week 4	IC-TAMRA	10 pg	19.4829	1.5264
week 4		1 pg	17.8260	0.9877
week 4		100 fg	16.6556	0.8792
week 4		NTC	12.6765	0.2611

Week	Target	Plasmid concentration per reaction	mean Ct n = 3	SD
week 6	IC-TAMRA	10 pg	18.9067	0.8923
week 6		1 pg	17.5203	0.8740
week 6		100 fg	16.6079	1.4922
week 6		NTC	12.2949	0.6223
Week	Target	Plasmid concentration per reaction	mean Ct n = 3	SD
week 8	IC-TAMRA	10 pg	19.3283	1.1386
week 8		1 pg	17.7890	1.0521
week 8		100 fg	16.7755	1.0269
week 8		NTC	12.6698	0.5952
Week	Target	Plasmid concentration per reaction	mean Ct n = 3	SD
week 10	IC-TAMRA	10 pg	19.0645	1.3189
week 10		1 pg	17.7839	1.2341
week 10		100 fg	16.5982	0.6837
week 10		NTC	12.4315	0.5656
Week	Target	Plasmid concentration per reaction	mean Ct n = 3	SD
week 12	IC-TAMRA	10 pg	17.0826	1.3656
week 12		1 pg	15.5669	1.1370
week 12		100 fg	14.4911	1.2066
week 12		NTC	12.3257	0.7695
Week	Target	Plasmid concentration per reaction	mean Ct n = 3	SD
week 13	IC-TAMRA	10 pg	15.4011	0.9992
week 13		1 pg	14.1443	0.7260
week 13		100 fg	13.5068	0.7354
week 13		NTC	11.6135	0.3048

Table S24. Raw data obtained with the *vt1*-CY5 probe during accelerated stability studies (product stored at 20°C).

20°C *vt1*-CY5 probe				
**Week**	**Target**	**Plasmid concentration per reaction**	**mean Ct n = 3**	SD
week 0	*vt1*-CY5	10 pg	5.8972	0.1624
week 0		1 pg	6.8234	0.2032
week 0		100 fg	7.6503	0.2367
week 0		NTC	ND	N/A
Week	Target	Plasmid concentration per reaction	mean Ct n = 3	SD
week 1	*vt1*-CY5	10 pg	5.8827	0.0843
week 1		1 pg	6.6617	0.1461
week 1		100 fg	7.4432	0.0437
week 1		NTC	21.7072	N/A
Week	Target	Plasmid concentration per reaction	mean Ct n = 3	SD

week 2	*vt1*-CY5	10 pg	5.9685	0.0331
week 2		1 pg	6.8197	0.0287
week 2		100 fg	7.9039	0.0503
week 2		NTC	30.9193	1.4702
Week	Target	Plasmid concentration per reaction	mean Ct n = 3	SD
week 3	*vt1*-CY5	10 pg	6.0419	0.1051
week 3		1 pg	7.0106	0.1204
week 3		100 fg	8.1325	0.1543
week 3		NTC	48.3909	N/A
Week	Target	Plasmid concentration per reaction	mean Ct n = 3	SD
week 4	*vt1*-CY5	10 pg	5.8650	0.0939
week 4		1 pg	6.6941	0.1338
week 4		100 fg	7.6390	0.0774
week 4		NTC	61.0000	N/A
Week	Target	Plasmid concentration per reaction	mean Ct n = 3	SD
week 6	*vt1*-CY5	10 pg	5.6922	0.0766
week 6		1 pg	6.3637	0.0578
week 6		100 fg	7.3282	0.1432
week 6		NTC	ND	#DIV/0!
Week	Target	Plasmid concentration per reaction	mean Ct n = 3	SD
week 8	*vt1*-CY5	10 pg	5.8799	0.0544
week 8		1 pg	6.7157	0.0962
week 8		100 fg	7.6439	0.1383
week 8		NTC	ND	N/A
Week	Target	Plasmid concentration per reaction	mean Ct n = 3	SD
week 10	*vt1*-CY5	10 pg	5.7019	0.1061
week 10		1 pg	6.4782	0.1369
week 10		100 fg	7.5093	0.1263
week 10		NTC	ND	N/A
Week	Target	Plasmid concentration per reaction	mean Ct n = 3	SD
week 12	*vt1*-CY5	10 pg	6.4290	0.2700
week 12		1 pg	7.5203	0.3877
week 12		100 fg	8.5342	0.3507
week 12		NTC	ND	N/A
Week	Target	Plasmid concentration per reaction	mean Ct n = 3	SD
week 13	*vt1*-CY5	10 pg	6.8600	0.4136
week 13		1 pg	7.5672	0.2746
week 13		100 fg	8.2655	0.1647
week 13		NTC	ND	N/A

Table S25. Raw data obtained with the *vt2*-FAM probe during accelerated stability studies (product stored at 20°C).

20°C *vt2*-FAM probe				
**Week**	**Target**	**Plasmid concentration per reaction**	**mean Ct n = 3**	SD
week 0	vt2-FAM	10 pg	5.7833	0.1700
week 0		1 pg	7.1467	0.1971
week 0		100 fg	8.4642	0.3362
week 0		NTC	51.7425	N/A
Week	Target	Plasmid concentration per reaction	mean Ct n = 3	SD
week 1	*vt2*-FAM	10 pg	6.1591	0.2261
week 1		1 pg	7.4079	0.2994
week 1		100 fg	8.4388	0.2196
week 1		NTC	48.9056	N/A
Week	Target	Plasmid concentration per reaction	mean Ct n = 3	SD
week 2	*vt2*-FAM	10 pg	6.1456	0.1326
week 2		1 pg	7.3474	0.1995
week 2		100 fg	8.7348	0.3045
week 2		NTC	41.9709	2.3233
Week	Target	Plasmid concentration per reaction	mean Ct n = 3	SD
week 3	*vt2*-FAM	10 pg	5.9413	0.0973
week 3		1 pg	7.2242	0.2232
week 3		100 fg	8.7930	0.2730
week 3		NTC	59.2467	N/A
Week	Target	Plasmid concentration per reaction	mean Ct n = 3	SD
week 4	*vt2*-FAM	10 pg	5.8784	0.2038
week 4		1 pg	7.0404	0.1676
week 4		100 fg	8.4077	0.2621
week 4		NTC	61.0000	N/A
Week	Target	Plasmid concentration per reaction	mean Ct n = 3	SD
week 6	*vt2*-FAM	10 pg	5.6375	0.1881
week 6		1 pg	6.7372	0.1630
week 6		100 fg	7.8716	0.3033
week 6		NTC	61.0000	#DIV/0!
Week	Target	Plasmid concentration per reaction	mean Ct n = 3	SD
week 8	*vt2*-FAM	10 pg	5.7591	0.1052
week 8		1 pg	6.9863	0.1220
week 8		100 fg	8.1946	0.2589
week 8		NTC	61.0000	N/A
Week	Target	Plasmid concentration per reaction	mean Ct n = 3	SD
week 10	*vt2*-FAM	10 pg	5.6195	0.1257
week 10		1 pg	6.7126	0.1666
week 10		100 fg	8.0301	0.0946
week 10		NTC	61.0000	#DIV/0!

Week	Target	Plasmid concentration per reaction	mean Ct n = 3	SD
week 12	*vt2*-FAM	10 pg	6.8098	0.2975
week 12		1 pg	8.2747	0.2565
week 12		100 fg	9.5771	0.4031
week 12		NTC	61.0000	N/A
Week	Target	Plasmid concentration per reaction	mean Ct n = 3	SD
week 13	*vt2*-FAM	10 pg	6.3861	0.2890
week 13		1 pg	7.4866	0.1017
week 13		100 fg	8.5957	0.2945
week 13		NTC	43.7804	4.8810

Table S26. Raw data obtained with the IC-TAMRA probe during accelerated stability studies (product stored at 37°C).

**37°C IC-TAMRA probe**				
**Week**	**Target**	**Plasmid concentration per reaction**	**mean Ct n = 3**	SD
week 0	IC-TAMRA	10 pg	19.5587	1.7049
week 0		1 pg	17.7458	1.3762
week 0		100 fg	16.4827	1.1691
week 0		NTC	13.0085	0.7622
Week	Target	Plasmid concentration per reaction	mean Ct n = 3	SD
week 1	IC-TAMRA	10 pg	19.7050	1.9195
week 1		1 pg	18.1826	1.6784
week 1		100 fg	17.2182	1.5500
week 1		NTC	12.9484	0.8482
Week	Target	Plasmid concentration per reaction	mean Ct n = 3	SD
week 2	IC-TAMRA	10 pg	16.8359	1.0731
week 2		1 pg	15.8016	0.7052
week 2		100 fg	14.6889	0.7189
week 2		NTC	12.1101	0.5322
Week	Target	Plasmid concentration per reaction	mean Ct n = 3	SD
week 3	IC-TAMRA	10 pg	17.3534	1.7945
week 3		1 pg	16.1436	1.5701
week 3		100 fg	15.0683	1.4043
week 3		NTC	11.8084	0.3955
Week	Target	Plasmid concentration per reaction	mean Ct n = 3	SD
week 4	IC-TAMRA	10 pg	18.3257	0.9578
week 4		1 pg	16.8815	1.2495
week 4		100 fg	15.7361	1.0675
week 4		NTC	12.1622	0.1489
Week	Target	Plasmid concentration per reaction	mean Ct n = 3	SD

week 6	IC-TAMRA	10 pg	18.8650	1.9194
week 6		1 pg	17.2741	1.2577
week 6		100 fg	16.3193	1.2163
week 6		NTC	12.1968	0.4505
Week	Target	Plasmid concentration per reaction	mean Ct n = 3	SD
week 8	IC-TAMRA	10 pg	19.2010	1.2117
week 8		1 pg	17.7081	0.9935
week 8		100 fg	16.5320	0.9401
week 8		NTC	12.5442	0.2574
Week	Target	Plasmid concentration per reaction	mean Ct n = 3	SD
week 10	IC-TAMRA	10 pg	18.0682	0.7800
week 10		1 pg	16.7065	0.6286
week 10		100 fg	15.7732	0.5976
week 10		NTC	12.2642	0.3597
Week	Target	Plasmid concentration per reaction	mean Ct n = 3	SD
week 12	IC-TAMRA	10 pg	15.7722	0.6358
week 12		1 pg	13.5396	0.5383
week 12		100 fg	11.5724	0.4343
week 12		NTC	11.2838	0.1884
Week	Target	Plasmid concentration per reaction	mean Ct n = 3	SD
week 13	IC-TAMRA	10 pg	15.4708	0.7470
week 13		1 pg	14.4195	0.8440
week 13		100 fg	13.8772	0.3603
week 13		NTC	11.5431	0.3348

Table S27. Raw data obtained with the *vt1*-CY5 probe during accelerated stability studies (product stored at 37°C).

**37°C *vt1*-CY5 probe**				
**Week**	**Target**	**Plasmid concentration per reaction**	**mean Ct n = 3**	SD
week 0	*vt1*-CY5	10 pg	5.8972	0.1624
week 0		1 pg	6.8234	0.2032
week 0		100 fg	7.6503	0.2367
week 0		NTC	ND	N/A
Week	Target	Plasmid concentration per reaction	mean Ct n = 3	SD
week 1	*vt1*-CY5	10 pg	5.9020	0.1268
week 1		1 pg	6.5970	0.1221
week 1		100 fg	7.4634	0.1293
week 1		NTC	31.0719	N/A
Week	Target	Plasmid concentration per reaction	mean Ct n = 3	SD
week 2	*vt1*-CY5	10 pg	6.0799	0.0460
week 2		1 pg	6.9760	0.0423

week 2		100 fg	8.1401	0.1148
week 2		NTC	ND	N/A
Week	Target	Plasmid concentration per reaction	mean Ct n = 3	SD
week 3	*vt1*-CY5	10 pg	5.9766	0.0658
week 3		1 pg	6.7836	0.0908
week 3		100 fg	7.9062	0.2124
week 3		NTC	49.8288	N/A
Week	Target	Plasmid concentration per reaction	mean Ct n = 3	SD
week 4	*vt1*-CY5	10 pg	5.7537	0.0651
week 4		1 pg	6.5759	0.0495
week 4		100 fg	7.6232	0.1756
week 4		NTC	ND	N/A
Week	Target	Plasmid concentration per reaction	mean Ct n = 3	SD
week 6	*vt1*-CY5	10 pg	5.8203	0.0694
week 6		1 pg	6.3953	0.1254
week 6		100 fg	7.3310	0.0928
week 6		NTC	28.0123	N/A
Week	Target	Plasmid concentration per reaction	mean Ct n = 3	SD
week 8	*vt1*-CY5	10 pg	5.9448	0.0622
week 8		1 pg	6.7788	0.1294
week 8		100 fg	7.6812	0.1262
week 8		NTC	ND	N/A
Week	Target	Plasmid concentration per reaction	mean Ct n = 3	SD
week 10	*vt1*-CY5	10 pg	5.9084	0.0509
week 10		1 pg	6.7343	0.1269
week 10		100 fg	7.7704	0.1519
week 10		NTC	ND	N/A
Week	Target	Plasmid concentration per reaction	mean Ct n = 3	SD
week 12	*vt1*-CY5	10 pg	7.3211	0.1094
week 12		1 pg	8.8872	0.2219
week 12		100 fg	11.7316	0.8676
week 12		NTC	ND	N/A
Week	Target	Plasmid concentration per reaction	mean Ct n = 3	SD
week 13	*vt1*-CY5	10 pg	6.6268	0.1562
week 13		1 pg	7.3678	0.1309
week 13		100 fg	8.2016	0.3494
week 13		NTC	52.8057	N/A

Table S28. Raw data obtained with the *vt2*-FAM probe during accelerated stability studies (product stored at 37°C).

37°C *vt2*-FAM probe				
**Week**	**Target**	**Plasmid concentration per reaction**	**mean Ct n = 3**	SD
week 0	*vt2*-FAM	10 pg	5.7833	0.1700
week 0		1 pg	7.1467	0.1971
week 0		100 fg	8.4642	0.3362
week 0		NTC	51.7425	N/A
Week	Target	Plasmid concentration per reaction	mean Ct n = 3	SD
week 1	*vt2*-FAM	10 pg	5.8276	0.1573
week 1		1 pg	6.8995	0.1133
week 1		100 fg	8.1290	0.1813
week 1		NTC	43.3925	N/A
Week	Target	Plasmid concentration per reaction	mean Ct n = 3	SD
week 2	*vt2*-FAM	10 pg	6.2172	0.2041
week 2		1 pg	7.4682	0.2225
week 2		100 fg	9.1988	0.3709
week 2		NTC	46.9998	5.1232
Week	Target	Plasmid concentration per reaction	mean Ct n = 3	SD
week 3	*vt2*-FAM	10 pg	6.4883	0.2759
week 3		1 pg	7.6921	0.3473
week 3		100 fg	9.0085	0.3112
week 3		NTC	ND	N/A
Week	Target	Plasmid concentration per reaction	mean Ct n = 3	SD
week 4	*vt2*-FAM	10 pg	5.3292	0.2407
week 4		1 pg	6.6653	0.3142
week 4		100 fg	8.0193	0.3479
week 4		NTC	ND	N/A
Week	Target	Plasmid concentration per reaction	mean Ct n = 3	SD
week 6	*vt2*-FAM	10 pg	5.9636	0.2251
week 6		1 pg	7.0028	0.1709
week 6		100 fg	8.2082	0.3498
week 6		NTC	56.2591	#DIV/0!
Week	Target	Plasmid concentration per reaction	mean Ct n = 3	SD
week 8	*vt2*-FAM	10 pg	5.6129	0.1845
week 8		1 pg	6.7468	0.1029
week 8		100 fg	7.9299	0.1505
week 8		NTC	38.4761	3.1290
Week	Target	Plasmid concentration per reaction	mean Ct n = 3	SD
week 10	*vt2*-FAM	10 pg	5.7580	0.0965
week 10		1 pg	6.9078	0.0905
week 10		100 fg	8.2817	0.1722
week 10		NTC	ND	N/A

Week	Target	Plasmid concentration per reaction	mean Ct n = 3	SD
week 12	*vt2*-FAM	10 pg	7.5211	0.1599
week 12		1 pg	9.0699	0.3485
week 12		100 fg	11.3403	0.6825
week 12		NTC	51.9428	5.2650
Week	Target	Plasmid concentration per reaction	mean Ct n = 3	SD
week 13	*vt2*-FAM	10 pg	6.3492	0.1123
week 13		1 pg	7.4099	0.1580
week 13		100 fg	8.3958	0.2164
week 13		NTC	36.6701	18.3147

Table S29. Raw data obtained with the IC-TAMRA probe during accelerated stability studies (product stored at 50°C).

**50°C IC-TAMRA probe**				
**Week**	**Target**	**Plasmid concentration per reaction**	**mean Ct n = 3**	SD
week 0	IC-TAMRA	10 pg	19.5587	1.7049
week 0		1 pg	17.7458	1.3762
week 0		100 fg	16.4827	1.1691
week 0		NTC	13.0085	0.7622
Week	Target	Plasmid concentration per reaction	mean Ct n = 3	SD
week 1	IC-TAMRA	10 pg	16.5772	1.0361
week 1		1 pg	15.5347	1.2544
week 1		100 fg	14.7829	0.9687
week 1		NTC	12.1699	0.6764
Week	Target	Plasmid concentration per reaction	mean Ct n = 3	SD
week 2	IC-TAMRA	10 pg	18.2194	2.7515
week 2		1 pg	16.5422	2.9436
week 2		100 fg	15.3283	2.6789
week 2		NTC	12.3502	1.4222
Week	Target	Plasmid concentration per reaction	mean Ct n = 3	SD
week 3	IC-TAMRA	10 pg	16.3728	1.2418
week 3		1 pg	15.3051	1.3564
week 3		100 fg	14.4253	1.1269
week 3		NTC	11.6073	0.6970
Week	Target	Plasmid concentration per reaction	mean Ct n = 3	SD
week 4	IC-TAMRA	10 pg	18.3257	0.9578
week 4		1 pg	16.8815	1.2495
week 4		100 fg	15.7361	1.0675
week 4		NTC	12.1622	0.1489
Week	Target	Plasmid concentration per reaction	mean Ct n = 3	SD

week 6	IC-TAMRA	10 pg	18.4319	1.6443
week 6		1 pg	16.6677	1.0309
week 6		100 fg	15.5978	1.2818
week 6		NTC	12.0714	0.3697
Week	Target	Plasmid concentration per reaction	mean Ct n = 3	SD
week 8	IC-TAMRA	10 pg	18.4094	1.7392
week 8		1 pg	16.5069	1.2875
week 8		100 fg	15.3123	1.4296
week 8		NTC	11.8673	0.8429
Week	Target	Plasmid concentration per reaction	mean Ct n = 3	SD
week 10	IC-TAMRA	10 pg	17.5193	1.4711
week 10		1 pg	15.9574	1.3190
week 10		100 fg	14.8884	1.2287
week 10		NTC	11.9081	0.7847
Week	Target	Plasmid concentration per reaction	mean Ct n = 3	SD
week 12	IC-TAMRA	10 pg	17.9763	1.7681
week 12		1 pg	16.3671	1.4913
week 12		100 fg	15.2052	1.2606
week 12		NTC	11.8875	0.5027
Week	Target	Plasmid concentration per reaction	mean Ct n = 3	SD
week 13	IC-TAMRA	10 pg	15.0363	0.2248
week 13		1 pg	14.7636	0.7120
week 13		100 fg	13.6550	0.2453
week 13		NTC	11.4655	0.2621

Table S30. Raw data obtained with the *vt1*-CY5 probe during accelerated stability studies (product stored at 50°C).

50°C *vt1*-CY5 probe				
**Week**	**Target**	**Plasmid concentration per reaction**	**mean Ct n = 3**	SD
week 0	*vt1*-CY5	10 pg	5.8972	0.1624
week 0		1 pg	6.8234	0.2032
week 0		100 fg	7.6503	0.2367
week 0		NTC	ND	N/A
Week	Target	Plasmid concentration per reaction	mean Ct n = 3	SD
week 1	*vt1*-CY5	10 pg	5.9786	0.0609
week 1		1 pg	6.8548	0.1783
week 1		100 fg	7.6845	0.2080
week 1		NTC	39.0962	10.0948
Week	Target	Plasmid concentration per reaction	mean Ct n = 3	SD
week 2	*vt1*-CY5	10 pg	6.1852	0.2431
week 2		1 pg	7.3367	0.2919

week 2		100 fg	8.4174	0.3248
week 2		NTC	44.0560	N/A
Week	Target	Plasmid concentration per reaction	mean Ct n = 3	SD
week 3	*vt1*-CY5	10 pg	5.8880	0.0763
week 3		1 pg	6.6640	0.1567
week 3		100 fg	7.5926	0.1901
week 3		NTC	46.6447	14.0263
Week	Target	Plasmid concentration per reaction	mean Ct n = 3	SD
week 4	*vt1*-CY5	10 pg	5.7537	0.0651
week 4		1 pg	6.5759	0.0495
week 4		100 fg	7.6232	0.1756
week 4		NTC	ND	N/A
Week	Target	Plasmid concentration per reaction	mean Ct n = 3	SD
week 6	*vt1*-CY5	10 pg	5.9927	0.0419
week 6		1 pg	6.7656	0.1321
week 6		100 fg	7.7025	0.2152
week 6		NTC	52.5951	N/A
Week	Target	Plasmid concentration per reaction	mean Ct n = 3	SD
week 8	*vt1*-CY5	10 pg	6.0044	0.0875
week 8		1 pg	6.7699	0.1331
week 8		100 fg	7.6588	0.2422
week 8		NTC	ND	N/A
Week	Target	Plasmid concentration per reaction	mean Ct n = 3	SD
week 10	*vt1*-CY5	10 pg	6.1179	0.0871
week 10		1 pg	6.9654	0.1302
week 10		100 fg	7.9026	0.2197
week 10		NTC	ND	N/A
Week	Target	Plasmid concentration per reaction	mean Ct n = 3	SD
week 12	*vt1*-CY5	10 pg	6.2121	0.1065
week 12		1 pg	7.0629	0.0916
week 12		100 fg	8.0246	0.2078
week 12		NTC	ND	N/A
Week	Target	Plasmid concentration per reaction	mean Ct n = 3	SD
week 13	*vt1*-CY5	10 pg	7.2444	0.1595
week 13		1 pg	7.6395	0.1238
week 13		100 fg	8.6666	0.2985
week 13		NTC	55.9312	N/A

Table S31. Raw data obtained with the *vt2*-FAM probe during accelerated stability studies (product stored at 50°C).

**50°C *vt2*-FAM probe**

**Week**	**Target**	**Plasmid concentration per reaction**	**mean Ct n = 3**	SD
week 0	*vt2*-FAM	10 pg	5.7833	0.1700
week 0		1 pg	7.1467	0.1971
week 0		100 fg	8.4642	0.3362
week 0		NTC	51.7425	N/A
Week	Target	Plasmid concentration per reaction	mean Ct n = 3	SD
week 1	*vt2*-FAM	10 pg	6.4414	0.1470
week 1		1 pg	7.4978	0.4471
week 1		100 fg	8.5719	0.3621
week 1		NTC	37.7400	N/A
Week	Target	Plasmid concentration per reaction	mean Ct n = 3	SD
week 2	*vt2*-FAM	10 pg	6.2382	0.0716
week 2		1 pg	7.1014	0.3760
week 2		100 fg	8.5039	0.6234
week 2		NTC	54.1593	N/A
Week	Target	Plasmid concentration per reaction	mean Ct n = 3	SD
week 3	*vt2*-FAM	10 pg	6.2097	0.2660
week 3		1 pg	7.3833	0.3928
week 3		100 fg	8.6295	0.4083
week 3		NTC	46.3125	10.1665
Week	Target	Plasmid concentration per reaction	mean Ct n = 3	SD
week 4	*vt2*-FAM	10 pg	5.2283	0.2281
week 4		1 pg	6.5515	0.3099
week 4		100 fg	7.9022	0.3438
week 4		NTC	ND	N/A
Week	Target	Plasmid concentration per reaction	mean Ct n = 3	SD
week 6	*vt2*-FAM	10 pg	5.2017	0.3206
week 6		1 pg	6.3392	0.1836
week 6		100 fg	7.6245	0.2257
week 6		NTC	53.3301	4.7348
Week	Target	Plasmid concentration per reaction	mean Ct n = 3	SD
week 8	*vt2*-FAM	10 pg	6.0911	0.2080
week 8		1 pg	7.3172	0.2236
week 8		100 fg	8.5313	0.3726
week 8		NTC	31.4032	N/A
Week	Target	Plasmid concentration per reaction	mean Ct n = 3	SD
week 10	*vt2*-FAM	10 pg	6.0058	0.1982
week 10		1 pg	7.2267	0.2759
week 10		100 fg	8.6388	0.2808
week 10		NTC	ND	N/A
Week	Target	Plasmid concentration per reaction	mean Ct n = 3	SD

week 12	*vt2*-FAM	10 pg	6.4592	0.2989
week 12		1 pg	7.6503	0.3717
week 12		100 fg	8.9582	0.4828
week 12		NTC	45.1591	8.4890
Week	Target	Plasmid concentration per reaction	mean Ct n = 3	SD
week 13	*vt2*-FAM	10 pg	6.8087	0.1342
week 13		1 pg	7.8071	0.1765
week 13		100 fg	9.1012	0.2794
week 13		NTC	57.8841	N/A

Table S32. Raw data used to calculate the Pearson correlation coefficient between the % of false positive results and storage temperature of the MAST ISOPLEX® *VTEC* kit. The analysis was performed with Minitab® Statistical Software v18 (Minitab®, LLC.).

Storage temperature	% of false positive results	Specificity
4°C	3.33	96.67
20°C	5	95
37°C	6.66	93.34
50°C	11.6	88.4

Pearson correlation = 0.94.

p-value = 0.06.
